# Evidence of somatic cell reprogramming toward embryogenic competence revealed through morphoanatomical and biochemical transitions in *Piper aduncum* L.

**DOI:** 10.1007/s00709-026-02221-x

**Published:** 2026-05-30

**Authors:** Natália Barros de Souza, Rennan Oliveira Meira, Jonny Everson Scherwinski-Pereira

**Affiliations:** 1 Department of Forest Engineering, University of Brasília, Brasília, 70910- 900 Brazil; 2 FAPDF/Embrapa Postdoctoral Fellowship Program, Embrapa Genetic Resources and Biotechnology, Brasília, Brazil; 3 Embrapa Genetic Resources and Biotechnology, Av. W5 Norte (final), Brasília, DF 70770-917 Brazil

**Keywords:** Piperaceae, Dillapiole, Somatic embryogenesis, Embryogenic competence, Redox metabolism, Clonal propagation

## Abstract

Somatic embryogenesis represents a well-established example of cellular plasticity in plants, allowing differentiated somatic cells to revert to a totipotent state and regenerate whole plants. In *Piper aduncum* L., a species of high economic value due to its dillapiole-rich essential oil, large-scale propagation remains limited by inefficient regeneration systems. This study provides integrative evidence of somatic cell reprogramming toward embryogenic competence from adult leaf explants cultured under controlled in vitro conditions. Morphoanatomical analyses revealed that reprogramming began within three days of culture, characterized by hypertrophic nuclei, dense cytoplasm, and perivascular clusters of small isodiametric cells acting as embryogenic niches. Sequential histological and histochemical changes accompanied the transition from callus proliferation to organized somatic embryos, culminating in cotyledonary structures exhibiting all primary meristems. The growth curve of embryogenic calli revealed five physiological phases, with the linear phase (28–49 days) corresponding to the optimal window for subculture and somatic embryo differentiation. Biochemical profiling indicated dynamic redox modulation, marked by transient peaks in antioxidant enzyme activity (SOD, CAT, APX, POD) and controlled lipid peroxidation, suggesting that oxidative signaling functions as a permissive factor during embryogenic induction and differentiation. Together, these findings demonstrate that somatic embryogenesis in *P. aduncum* arises from a coordinated sequence of cellular and physiological reprogramming events in adult tissues, providing new insights into the developmental plasticity and redox regulation underlying plant regeneration.

## Introduction

The genus *Piper* (Piperaceae) comprises approximately 700 species distributed worldwide, about 170 of which occur in Brazil (Souza and Lorenzi 2012). Many *Piper* species are traditionally used in folk medicine and hold high economic value. The production of essential oils, a hallmark of the genus, supports applications in the condiment, pharmaceutical, and biocontrol industries (Durofil et al. 2021; Pérez et al. 2022; Fazolin et al. 2022). Among them, *Piper aduncum* L. stands out for its essential oil rich in phenylpropanoids, particularly dillapiole, the major compound in Amazonian chemotypes (Fazolin et al. 2006). Dillapiole exhibits strong bioactivity against agricultural pests such as fungi, insects, larvae, and mollusks (Fazolin et al. 2005, 2007, 2015, 2023; Volpe et al. 2016, 2018), positioning *P. aduncum* as a promising natural source for biopesticide development and as an alternative to synthetic agrochemicals for domestic and international markets. However, the commercial potential of *P. aduncum* depends on the production of uniform, chemically stable genotypes. Studies on monoclonal cultivations indicate that clonal inheritance contributes to the stability of essential oil profiles under controlled conditions (Ramos et al. 2022), emphasizing the need for reliable clonal propagation methods. Seed propagation remains the most widely used and convenient approach for seedling production, but genetic segregation causes significant variation in essential oil composition and yield, hindering the establishment of homogeneous plantations. Vegetative propagation via stem cuttings (Dousseau 2009; Gomes and Krinski 2018; Susanto et al. 2019; Ferriani et al. 2019) offers partial clonality but suffers from low productivity and reduced rooting capacity as donor plants age (Cid and Teixeira 2010).

Micropropagation therefore represents an effective alternative for the large-scale multiplication of elite *Piper* genotypes with high dillapiole content (De Sousa et al. 2020). Among in vitro approaches, somatic embryogenesis (SE) is particularly promising for mass propagation once optimized for each genotype (Carvalho et al. 2006). Nevertheless, embryogenic responses are strongly influenced by explant origin, endogenous hormonal balance, and tissue physiological state (Capriotti et al. 2022; Silva-Cardoso et al. 2022). These factors are especially critical when explants are derived from greenhouse or field-grown plants, which typically show lower embryogenic competence than seedlings obtained through in vitro germination, as described in the pioneering SE protocol for *P. aduncum* (De Sousa et al. 2020). Although effective, that protocol still requires refinement to enable large-scale commercial application.

Advancing somatic embryogenesis systems requires understanding where and how cellular reprogramming begins. Morphoanatomical studies have shown that the acquisition of embryogenic competence is linked to clusters of small, isodiametric cells with dense cytoplasm and prominent nuclei/nucleoli, often originating near vascular bundles (Verdeil et al. 2001; Sané et al. 2006; Ulisses et al. 2010; Meira et al. 2019; Silva-Cardoso et al. 2019). Integrating such observations with callus growth kinetics allows the mapping of physiological phases and the identification of optimal subculture points (Nogueira et al. 2008). Beyond the cellular level, stress plays a pivotal role in triggering SE, modulating gene expression, hormonal signaling, and cellular competence. Controlled levels of abiotic stress, such as wounding, osmotic or thermal stimuli, can induce embryogenic transition, while excessive stress redirects metabolism toward defense pathways and inhibits totipotency (Zavattieri et al. 2010). In vitro culture itself imposes stress through excision, disinfection, and medium exposure, processes that lead to the accumulation of reactive oxygen species (ROS) (Nolan et al. 2006; Batool et al. 2019; Orłowska and Kępczyńska 2020). To balance ROS production and avoid oxidative damage, plants rely on enzymatic antioxidant systems such as superoxide dismutase (SOD), catalase (CAT), ascorbate peroxidase (APX), and peroxidase (POD), which regulate redox homeostasis and influence the acquisition of embryogenic competence (Knörzer et al. 1996; Teisseire and Guy 2000; Ahmad et al. 2010; Meena et al. 2024). Current evidence supports a model in which somatic embryogenesis arises from the interaction between competent cellular niches (morphological/anatomical) and a permissive redox window (biochemical), dynamically balanced by antioxidant activity.

To explore these interactions, the present study combines (i) time-course morphoanatomical analyses of explants and calli, (ii) callus growth kinetics to define physiological phases and subculture timing, and (iii) enzymatic and redox profiling (SOD, CAT, APX, POD, and MDA) as functional markers of stress modulation and embryogenic competence. The main objective was to elucidate the cellular and physiological processes underlying SE induction and development in *P. aduncum* from adult ex vitro leaves, providing a comprehensive framework to optimize propagation protocols and enable large-scale clonal multiplication of elite genotypes.

## Materials and methods

### Somatic embryogenesis in *Piper aduncum*

Apical leaves from adult *Piper aduncum* L. plants (accession A2080005) maintained at the Active Germplasm Bank of Embrapa Acre, Brazil (10°15′57″ S, 67°42′17″ W), were collected from greenhouse-grown plants. Species identification was confirmed at the Rio de Janeiro Botanical Garden, and a voucher specimen was deposited under number 8817. Authorization for access to Brazilian biodiversity was granted by IBAMA under permits 02001.050950/2011-61 (scientific research) and 02000.000460/2013-96 (bioprospection). Explants were surface-sterilized in 70% (v/v) ethanol for 2 min, followed by immersion in 0.75% (v/v) sodium hypochlorite solution prepared from commercial bleach containing 5–6% active chlorine (Start^®^) for 15 min. Explants were then rinsed three times with sterile distilled water, and leaf sections of approximately 1 cm² were placed with the abaxial surface in contact with the culture medium.

Embryogenic callus induction was carried out on basal MS medium (Murashige and Skoog 1962) supplemented with 30 g L⁻¹ sucrose (Sigma-Aldrich^®^), 2.5 g L⁻¹ Phytagel™ (Sigma-Aldrich^®^), 26.85 µM α-naphthaleneacetic acid (NAA), and 11.10 µM 6-benzylaminopurine (BAP). Cultures were maintained in darkness at 25 ± 2 °C for 45 days. This induction medium and culture conditions were established according to De Sousa et al. (2020). For somatic embryo differentiation, embryogenic calli were subsequently transferred to a modified MS-based medium supplemented with 30 g L⁻¹ sucrose, 2 g L⁻¹ polyvinylpyrrolidone (PVP-40), 2.5 g L⁻¹ Phytagel, 2.26 µM 2,4-dichlorophenoxyacetic acid (2,4-D), and 11.10 µM BAP. In this phase, the auxin NAA used by De Sousa et al. (2020) was replaced by 2,4-D, while the BAP concentration was maintained. Additional modifications, including medium composition and culture conditions for differentiation and maturation, were developed in the present study. Cultures remained on this medium for 30 days until the appearance of the first somatic embryos. Calli containing early-stage embryos (globular and torpedo stages) were then transferred to a maturation medium consisting of MS salts with 45 g L⁻¹ sucrose, 2 g L⁻¹ PVP-40, and 2 g L⁻¹ Phytagel, and maintained for 60 days to promote embryo development to the cotyledonary stage.

Germination of somatic embryos was induced on half-strength MS medium containing 45 g L⁻¹ sucrose and 1.44 µM gibberellic acid (GA₃), followed by plantlet development on hormone-free MS medium.

### Morphoanatomical analyses

Samples were collected at 0 days (leaf prior to inoculation) and at 3, 7, 14, 21, 30, 45, and 60 days of culture on induction medium. Additional samples were collected after 30 days in embryo differentiation medium and cotyledonary embryos after 60 days in maturation medium.

Samples were fixed in Karnovsky’s solution (Karnovsky 1965), rinsed in 0.05 M sodium cacodylate buffer (pH 7.2), dehydrated in a graded ethanol series, and embedded in historesin (Leica^®^, Heidelberg, Germany) according to the manufacturer’s instructions. Longitudinal and transverse sections were obtained using a manual rotary microtome (Leica^®^). For structural and histochemical characterization, sections were stained with 0.5% toluidine blue (O’Brien et al. 1964) to visualize general tissue organization and phenolic compounds. Starch was detected using Lugol’s reagent (Berlyn et al. 1976), and proteins were identified with Xylidine Ponceau (Vidal 1970). Images were captured under a Leica^®^ light microscope equipped with a digital acquisition system using LAS EZ v. 2.0 software.

### Callus growth curve

The callus growth curve was determined by recording fresh mass every seven days from day 0 to day 63 of culture on induction medium. Callus weight was calculated as the difference between the Petri dish weight containing callus plus medium and the weight of the Petri dish containing only the medium.

A total of ten weekly subcultures were performed. Each replicate consisted of five calli, and two independent replicates were used (*n* = 10). The growth percentage was calculated according to Smith (1992), using the formula: Growth (%) = ((ffw-ifw)/ifw)*100, where *ifw* represents the initial fresh weight and *ffw* the final fresh weight of the calli. The mean fresh weight at each time point was used to construct the growth curve.

### Biochemical and enzymatic characterization

Leaf explant samples were collected before and after surface sterilization, at 14, 30, and 45 days of culture on callus induction medium, and at 30 days on embryo differentiation medium. After collection, samples were rinsed in ultrapure water to remove medium residues, transferred to cryotubes, immediately frozen in liquid nitrogen, ground in porcelain mortars under liquid nitrogen, and stored at − 80 °C until analysis.

Lipid peroxidation was evaluated by quantifying malondialdehyde (MDA) through its reaction with thiobarbituric acid (TBARS), as described by Heath and Packer (1968). Antioxidant enzyme activities of superoxide dismutase (SOD), catalase (CAT), ascorbate peroxidase (APX), and peroxidase (POD) were determined spectrophotometrically and normalized to total soluble protein content, which was quantified prior to analysis. SOD activity was assayed according to Beauchamp and Fridovich (1971), based on inhibition of nitroblue tetrazolium (NBT) photoreduction at 560 nm. CAT activity was determined following Havir and McHale (1987), with modifications by Azevedo et al. (1998), by monitoring hydrogen peroxide (H₂O₂) decomposition at 240 nm. APX activity was evaluated using the method of Nakano and Asada (1981), based on the oxidation of ascorbic acid in the presence of H₂O₂, with absorbance measured at 290 nm. POD activity was determined according to Teisseire and Guy (2000), through the oxidation of guaiacol in the presence of H₂O₂, with the reaction product measured at 470 nm.

All spectrophotometric readings were performed with a Shimadzu UV-1800 UV-Vis spectrophotometer with glass or quartz cuvettes according to the wavelength requirements of each assay.

### Statistical analysis

Statistical analyses of the enzymatic activity data were conducted using R software (version 4.4.0). Data were expressed as mean ± standard error (SE) of three biological replicates, each in triplicate. Differences among treatments were evaluated by analysis of variance (ANOVA) followed by Tukey’s test (*p* ≤ 0.05).

## Results and discussion

### Morphogenesis of somatic embryogenesis in *Piper aduncum*

**Fig. 1:**
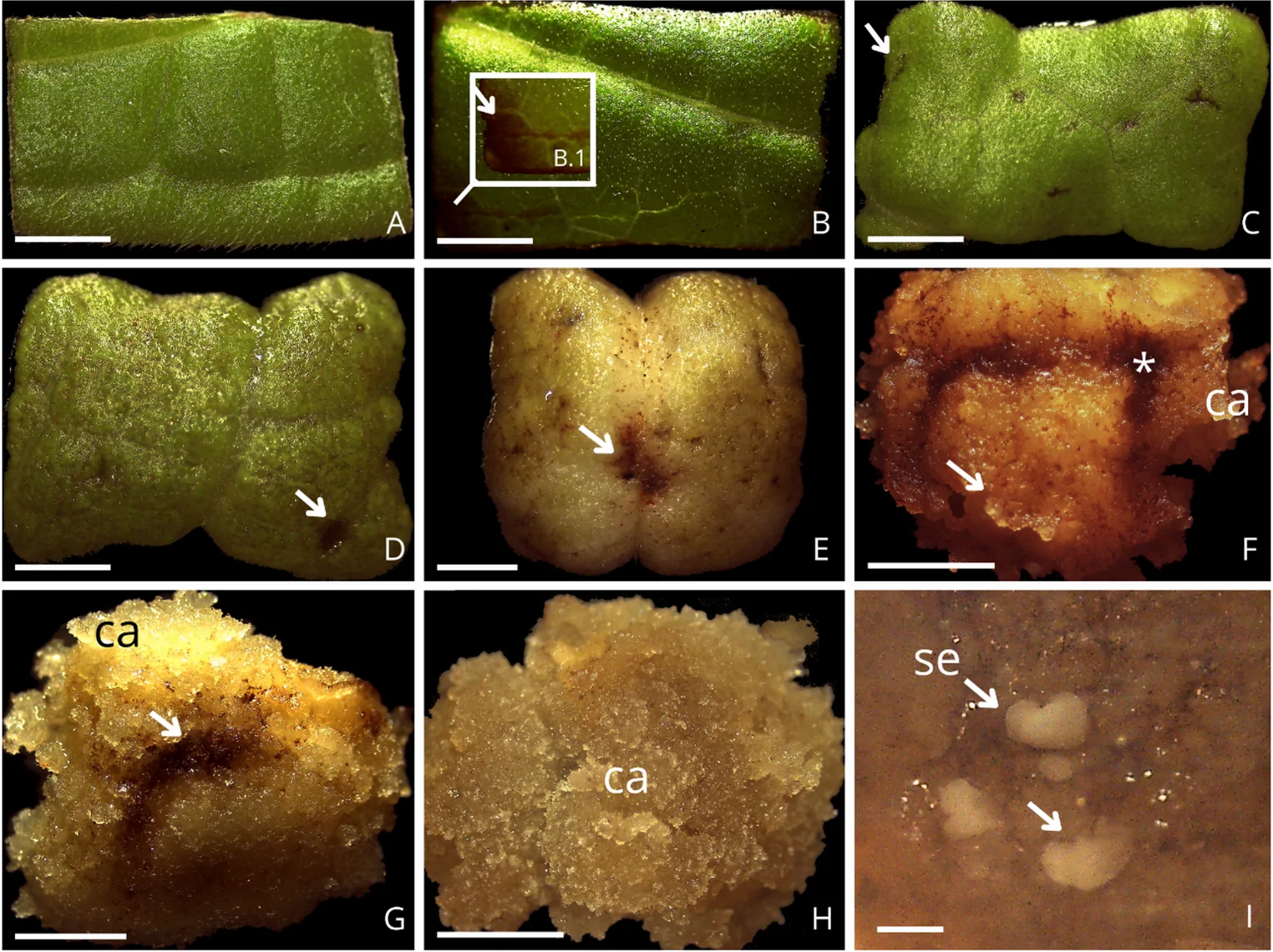
Morphological progression of callus formation and somatic embryo development from leaf explants of *Piper aduncum* L. obtained from greenhouse-grown adult plants. (**A**) Leaf explant at inoculation day. (**B**–B.1) Explant after 3 days of culture showing oxidized areas (arrow). (**C**) Explant after 7 days with undulated surface (arrow). (**D**) Explant after 14 days showing color change and onset of tissue oxidation (arrow). (**E**) Explant after 21 days with complete color transition and oxidized areas (arrow). (**F**) Explant after 30 days showing initial callus formation (arrow) and oxidized region (*). (**G**) Explant after 45 days exhibiting friable calli and oxidized areas (arrow). (**H**) Explant after 60 days with the entire surface covered by friable callus. (**I**) Emergence of somatic embryos (arrows) after 30 days on differentiation medium, located on the abaxial face of the calli. Abbreviations: ca., callus; se, somatic embryo. Bars = 2 mm (A–I)

Calli induced from leaf explants showed progressive morphological changes throughout 60 days of culture. Initially, the explants displayed a flat surface, green coloration, and the presence of trichomes (Fig. 1a). By the third day of culture, tissue oxidation was observed at the margins (Fig. 1b, b.1). After seven days, tissue swelling became evident, accompanied by irregularities along the edges and morphological changes, while the coloration remained predominantly green (Fig. 1c).

At 14 days, the explants exhibited expanded tissues with a slightly rough texture and a reduction in green coloration, which became opaque (Fig. 1d). A complete color transition occurred by 21 days, when the tissues thickened, turned yellowish, and displayed oxidized regions (Fig. 1e). After 30 days, the yellow coloration became more intense and uniform, with isolated oxidized areas. At this stage, the first friable structures appeared, mostly located at the explant periphery (Fig. 1f). After 45 days of culture, the quantity and distribution of these structures increased, showing a translucent yellowish appearance (Fig. 1g). By the end of the 60-day culture period, the explants were fully covered by friable calli with a yellow-brown translucent texture, indicating advanced morphogenetic progression (Fig. 1h).

The morphogenetic pattern observed in this study differed from that previously described for other *Piper* species. The callogenic response was characterized by diffuse swelling, gradual tissue color transition from green to yellowish-brown tones, and the formation of friable calli completely covering the explant surface after 60 days of culture. In *P. aduncum*, De Sousa et al. (2020) reported friable beige-to-white calli after 40 days of culture on MS medium supplemented with NAA and BAP, mainly along leaf veins and wound edges. Likewise, *P. hispidinervum* formed friable whitish calli after 30 days (De Sousa et al. 2022), while *P. longum* produced nodular embryogenic calli under 2,4-D/NAA and cytokinin treatments (Thapa et al. 2024). Such variations may result from differences in physiological age, genotype, and source material, as juvenile in vitro-derived explants often exhibit higher morphogenic plasticity than adult tissues (Merkle 1995; Martini et al. 2013; Ballester et al. 2016; Corredoira et al. 2019; Capriotti et al. 2022).

### Hormonal regulation and 2,4-D-dependent competence

The differentiation medium previously described for *Piper* species (De Sousa et al. 2020, 2022) was ineffective for the genotype evaluated in the present study, making it necessary to investigate alternative hormonal conditions capable of promoting somatic embryo development from embryogenic calli. Considering that the explants used in the present study were obtained from adult ex vitro plants, physiological maturity and genotype-dependent responses were considered potential factors influencing morphogenic competence under in vitro conditions.

**Fig. 2:**
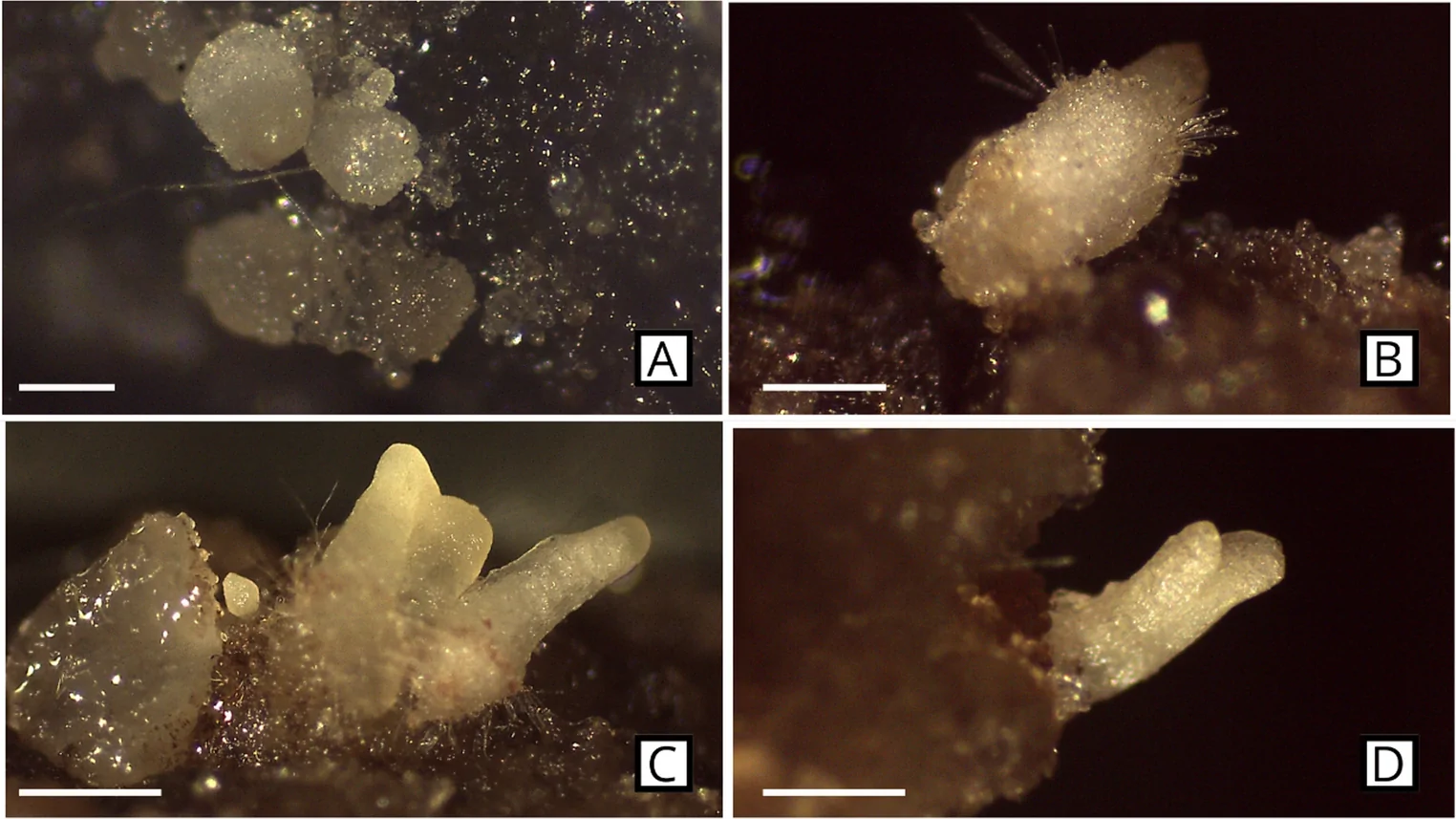
Development of somatic embryos of *Piper aduncum* from embryogenic calli obtained from leaf tissues. (**A**–**B**) Somatic embryos after 30 days on differentiation medium, exhibiting morphology consistent with the globular (**A**) and torpedo (**B**) stages. (**C**–**D**) Progression of embryogenic structures after an additional 30 days on maturation medium, showing increased size of somatic embryos reaching the cotyledonary stage. Scale bars = 0.5 mm (a, b); 2.5 mm (**C**, **D**)

After 30 days in differentiation medium containing 2.26 µM 2,4-D and 11.10 µM BAP, the first somatic embryos at globular, cordiform and cotyledonary stages were observed (Figs. 1i and 2a-d). The formation of embryos under these conditions suggests that the replacement of NAA with 2,4-D favored the maintenance of embryogenic competence and promoted cellular reprogramming required for somatic embryo differentiation. Although indirect somatic embryogenesis typically requires reducing or removing 2,4-D to promote differentiation and maturation, genotype-dependent exceptions have been reported. Short and controlled exposure to 2,4-D can maintain proembryogenic identity while endogenous IAA biosynthesis and redistribution re-establish cellular polarity (Horstman et al. 2017; Fehér 2019; Elhiti and Stasolla 2022; Li et al. 2022).

The dual function of 2,4-D, combining auxin signaling and mild stress induction, is considered crucial for competence acquisition, as it sustains a hormonal threshold until endogenous control resumes (Karami et al. 2023; Elhiti and Stasolla 2022). This mechanism aligns with the *Piper* system, where NAA + BAP supports embryogenic competence (De Sousa et al. 2020, 2022). In adult *ex vitro* tissues, a short 2,4-D + BAP pulse may synchronize embryogenic cell populations, guiding development toward globular and torpedo stages, provided hormone levels are subsequently reduced to avoid maturation blockage (Horstman et al. 2017; Garcia et al. 2019).

### Embryo maturation and germination

After 60 days in maturation medium, both embryo number and size increased markedly (Fig. 2): Embryogenic calli contained on average seven embryos (0.4 mm in length) after differentiation and 19 embryos (3.55 mm in length) after maturation. This result indicated that the maturation conditions promoted substantial embryo development and progression to advanced stages.

Maturation is a critical phase in somatic embryogenesis, requiring medium optimization to ensure viability and morphogenesis. Sucrose acts not only as a carbon and energy source but also modulates osmotic potential and solute transport (George et al. 2008; Espinoza et al. 2017; Campos-Boza et al. 2022; De Paiva Neto and Otoni 2003). Consistent with previous findings (Maciel et al. 2003; Ponnusamy 2012; Cao et al. 2022; Meira et al. 2025), sucrose supplementation strongly enhanced maturation efficiency.

**Fig. 3:**
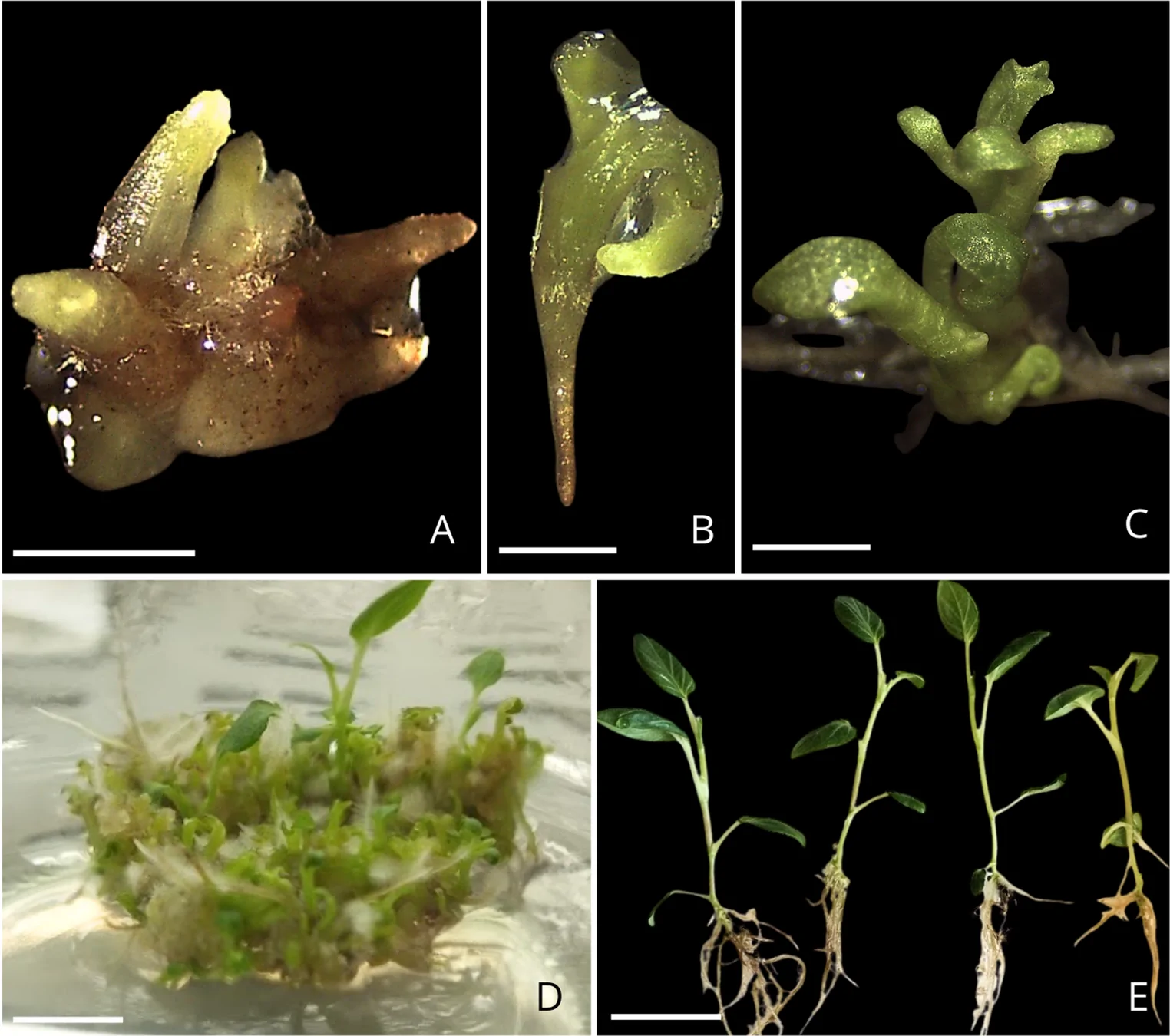
Germination of somatic embryos and plantlets development of *Piper aduncum*. (**A**) Somatic embryo after 5 days in germination medium containing 0.5 mg·L⁻¹ GA₃. (**B**) Plantlets with visible shoot and root tissues after 10 days in germination medium. (**C**–**D**) Formation and elongation of plantlets after 30 days in growth regulator-free development medium. (**E**) Vigorous plantlets with a well-developed root system after 60 days of culture. Scale bars = 2 cm (**E**); 0.5 cm (**A**, **D**); 3 mm (**C**); 2 mm (**B**)

Somatic embryo germination began between the 5th and 10th day of culture on GA₃ medium, characterized by radicle protrusion and shoot emergence (Fig. 3a–b). GA₃ promotes dormancy release by activating genes involved in growth inhibitor degradation (Montero-Córtes et al. 2011; Fehér 2015). Following germination, plantlets developed further in hormone-free MS medium, showing normal shoot and root growth after 30 days and full morphophysiological development suitable for acclimatization after 60 days (Fig. 3c–e).

### Ontogeny of somatic embryogenesis in *Piper aduncum*: morphoanatomical and histochemical analyses

#### Leaf anatomy and structural characterization

**Fig. 4:**
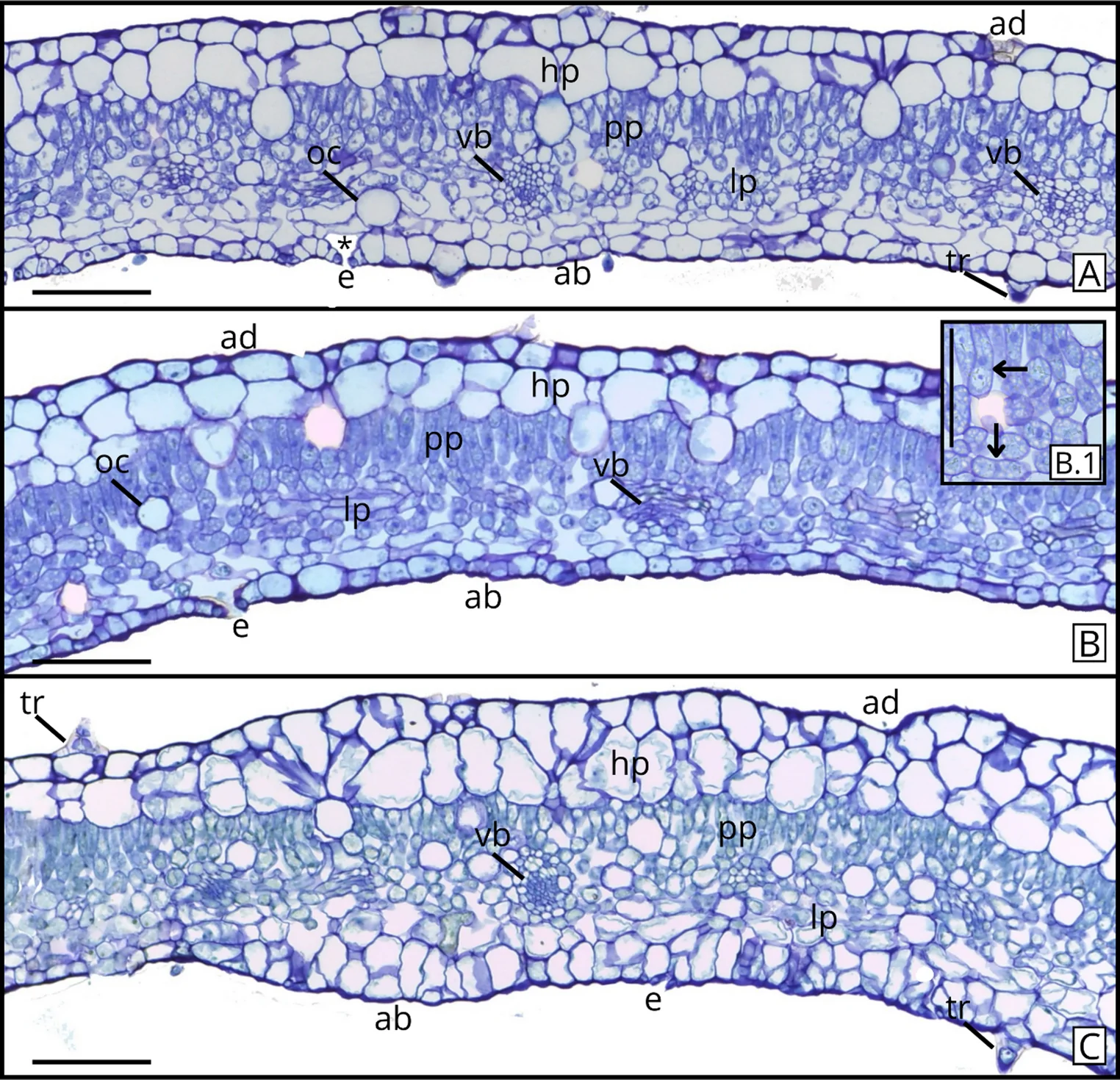
Anatomical characteristics of *Piper aduncum* leaf explants at 0, 3, and 7 days after inoculation on callus induction medium for somatic embryogenesis. (**A**) Leaf blade at the day of inoculation. (**B**) Leaf blade after 3 days of culture showing mesophyll cells with prominent nuclei, dense cytoplasm, and few intercellular spaces. (B.1) Detail of mesophyll cells undergoing division, exhibiting visible nuclei and dense cytoplasm (arrows). (**C**) General view of the leaf blade after 7 days of culture showing enlargement of epidermal and hypodermal cells, some with cytoplasmic retraction; parenchyma cells with signs of oxidation (greenish coloration); and vascular bundles with proliferative activity. Abbreviations: ab, abaxial epidermis; ad, adaxial epidermis; oc, oil cell; e, stoma; vb, vascular bundle; hp, hypodermis; pl, spongy parenchyma; pp, palisade parenchyma; tr, trichome. Scale bars = 200 μm (A–F); 50 μm (B.1)

The leaf blade epidermis of *Piper aduncum* is uniseriate on both adaxial and abaxial surfaces, composed of circular to rectangular cells with clearly defined cell walls. The adaxial surface contains a hypodermis (Fig. 4a). Although Gogosz et al. (2012) described the epidermis as multiseriate, other authors, including Maciel et al. (2014) and Nakamura et al. (2015), classified it as uniseriate with a multiple hypodermal layer. In this study, the subepidermal layer on the adaxial face is referred to as a hypodermis, based on ontogenetic evidence indicating its origin from the ground meristem (Nakamura et al. 2015).

The leaves are hypostomatic, a typical feature of the Piperaceae family (Judd et al. 1999), and exhibit trichomes on both surfaces of the lamina (Fig. 4, c). These are tectorial or glandular, multicellular and uniseriate trichomes (Maciel et al. 2014), also reported in *P. hispidinervum*. In non-cultured leaves (time 0), the mesophyll displays discrete, peripherally located nuclei and lightly stained cytoplasm (Fig. 4a), consistent with fully differentiated tissue under basal metabolic activity.

#### Early reprogramming events (3–7 days): nuclear activation and ROS signaling

After three days of culture (Fig. 4b), mesophyll cells showed increased nuclear size and prominence, denser cytoplasm, and small clear regions indicative of vacuolation in reorganization. Some epidermal and hypodermal cells exhibited conspicuous nuclei with visible nucleoli. These cytological features are classical histological markers of cellular reprogramming and embryogenic competence acquisition (Verdeil et al. 2001, 2007; Fehér 2015; Silva-Cardoso et al. 2019), indicating early activation of the embryogenic program. Similar behavior was reported in *Coffea arabica* cv. Mundo Novo, in which cell division began around day 8 of culture, mainly in the spongy parenchyma, with cells showing dense cytoplasm, prominent nuclei, and few intercellular spaces (Ferrari et al. 2021).

By day 7 (Fig. 4c), some hypodermal cells displayed irregular cytoplasmic retraction (plasmolysis or stress response), whereas the mesophyll exhibited a greener hue consistent with phenolic oxidation. This response likely reflects reactive oxygen species (ROS) accumulation, whose modulation is a key determinant for the acquisition or loss of embryogenic competence (Luo et al. 2024). In vascular bundles, clusters of dividing cells, forming incipient callogenic masses, appeared morphologically similar to those reported in palms (Silva-Cardoso et al. 2022). Anatomically, the precise origin of these clusters cannot be established; they may derive from either perivascular parenchyma divisions or proliferation of procambial/stem-cell-like cells associated with the procambium (Wang et al. 2011; Rose 2016; Silva-Cardoso et al. 2022).

#### Activation of vascular niches and pluripotent domains (14–21 days)

**Fig. 5:**
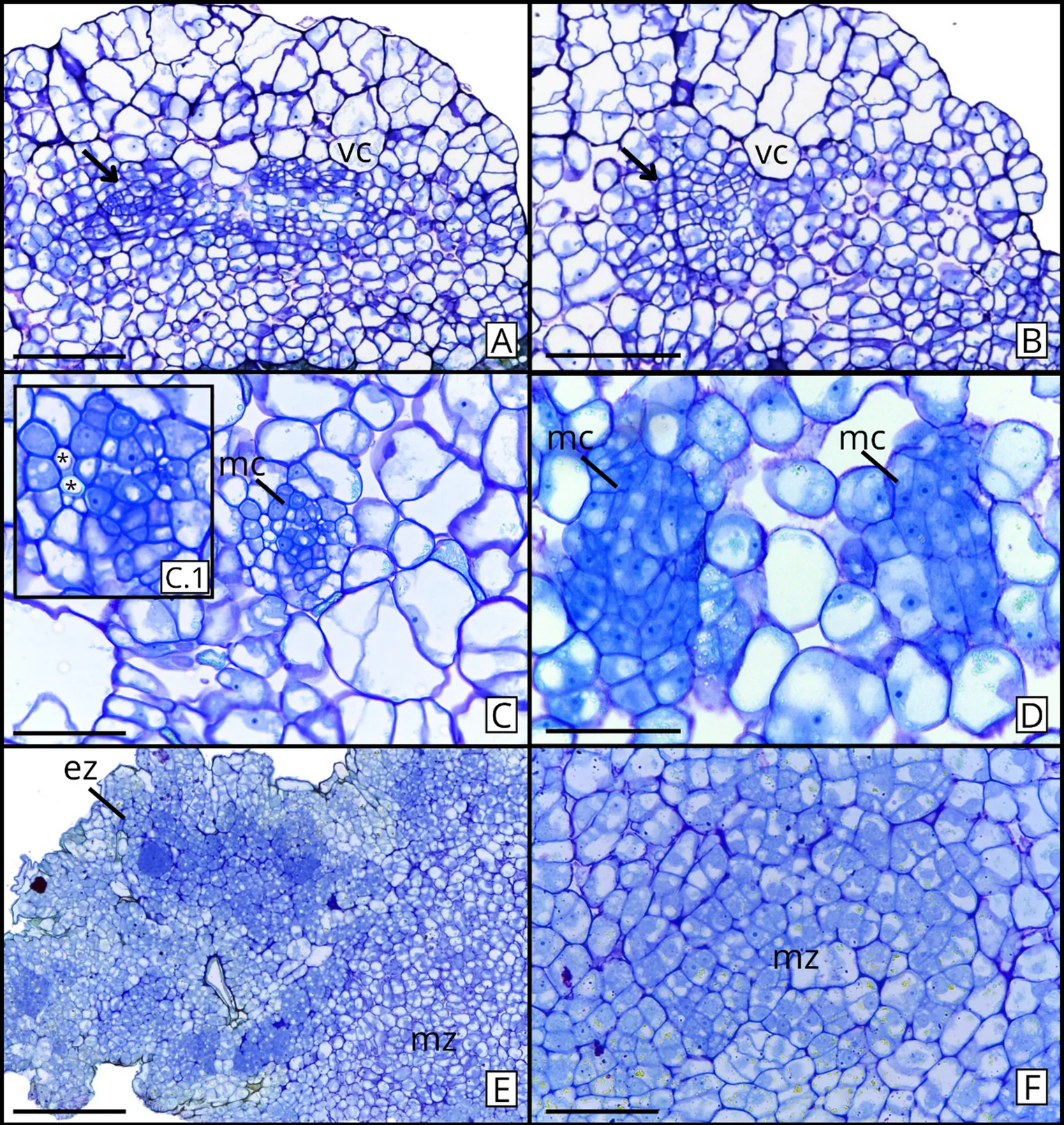
Anatomical characteristics of *Piper aduncum* leaf explants (**A**–**D**) and embryogenic calli (**E**–**F**) after 14, 21, and 30 days of culture on callus induction medium for somatic embryogenesis. (**A**–**B**) Leaf explant after 14 days of culture showing cellular reorganization in the mesophyll, with vacuolated cells at the periphery and partially preserved vascular bundles surrounded by smaller cells with dense cytoplasm and prominent nuclei (arrow). (**C**–**D**) Mesophyll region after 21 days of culture showing the formation of a meristematic center associated with a vascular bundle. (C.1) Detail of the developing meristematic center with xylem remnants (asterisks). (**E**–**F**) Callus after 30 days of culture showing compact cell clusters with dense cytoplasm and prominent nuclei, indicative of proliferative activity and embryogenic potential. Abbreviations: ez, embryogenic zone; mc, meristematic center; mz, meristematic zone; pc, primary callus; vc, vacuolated cell. Scale bars = 200 μm (**E**); 50 μm (**A**–**D**, **F**)

After 14 days of culture (Fig. 5a–b), partial loss of mesophyll and epidermal organization was observed. Near the epidermis, large vacuolated cells predominated, some forming small clusters with thickened walls, suggesting low reprogramming potential. In contrast, the central region adjacent to partially preserved vascular bundles showed intense proliferation of small isodiametric cells with dense cytoplasm, evident nuclei, and prominent nucleoli, which are typical of early meristematic activity. These findings suggest that vascular bundles act as preferential niches for cellular reprogramming during callus induction. This phase coincides with the “reprogramming window” (0–14 days) described for other species, characterized by activation of genes related to cell division, wall remodeling, and auxin pathways (Park et al. 2023).

Recent evidence indicates that procambial or perivascular cell reprogramming leads to a pluripotent state characterized by high plasticity and redifferentiation potential (Guo et al. 2023). Under appropriate hormonal and epigenetic conditions, this state can evolve to totipotency, expressed by somatic embryo formation (Sugimoto et al. 2011). In *P. tuberculatum* and *P. permucronatum*, embryogenic callus formation occurs around 14 days under 2,4-D and BAP (Guimarães 2015; Santos and Nogueira 2018). The slightly delayed response observed here may reflect *P. aduncum*’s specific sensitivity to the NAA + BAP combination and the physiological state of the explants, suggesting interspecific and explant-source variability.

#### Meristematic zone formation and competence acquisition (21–30 days)

After three weeks, meristematic zones emerged, composed of small isodiametric cells with dense cytoplasm (Fig. 5c), associated with small-caliber vascular bundles retaining xylem remnants (Fig. 5c.1). Compact meristematic centers developed simultaneously, consisting of dense cell clusters with prominent nuclei derived from vascular regions (Fig. 4d). This origin aligns with reports that vascular and perivascular tissues act as reprogramming niches during somatic embryogenesis in *P. aduncum* (De Sousa et al. 2020) and other plants (Verdeil et al. 2001; Jiménez 2001; Slesak et al. 2005; Fehér 2015; Silva-Cardoso et al. 2022).

According to Kwaaitaal and De Vries (2007), Somatic Embryogenesis Receptor Kinase 1 (SERK1) is expressed in procambial cells of vascular bundles, confirming their pluripotent nature and potential to acquire totipotency under specific hormonal conditions (Fukuda 2004). Procambial cells, which exhibit stem-cell-like behavior, are capable of self-renewal and differentiation into specialized cell types (Alison et al. 2002; Rippon and Bishop 2004) and are thus classified as pluripotent stem cells (Mahonen et al. 2006). SERK1 expression supports their role in embryogenic competence acquisition.This heterogeneity, where some cells retaining low competence while others undergo embryogenic reprogramming, is typical of calli under induction (Verdeil et al. 2001; Fehér 2015; Silva-Cardoso et al. 2019). Previous studies on *P. aduncum* and *P. hispidinervum* reported similar patterns, though embryogenic regions were not evident (De Sousa et al. 2020, 2022). Differences likely stem from explant physiology, as prior work used juvenile tissues from in vitro plants.

Juvenile explants, especially those pre-adapted to in vitro culture, exhibit greater cellular plasticity, higher metabolic activity, and lower phenolic accumulation, enhancing regenerative capacity (Fehér 2005; Pinheiro et al. 2012; Zurita-Valencia et al. 2014; Maciel et al. 2014). Conversely, adult *ex vitro* tissues require readaptation, showing heterogeneous responses that may necessitate rejuvenation strategies (von Aderkas and Bonga 2000). The present results demonstrate that even adult *ex vitro* material can trigger somatic embryogenesis when hormonal and environmental conditions are properly balanced. The NAA (26.85 µM) + BAP (11.10 µM) medium effectively induced embryogenic pathways in both juvenile and adult explants, with adult tissues responding more slowly and heterogeneously.

#### Embryogenic callus differentiation and histochemical evidence (30–45 days)

**Fig. 6:**
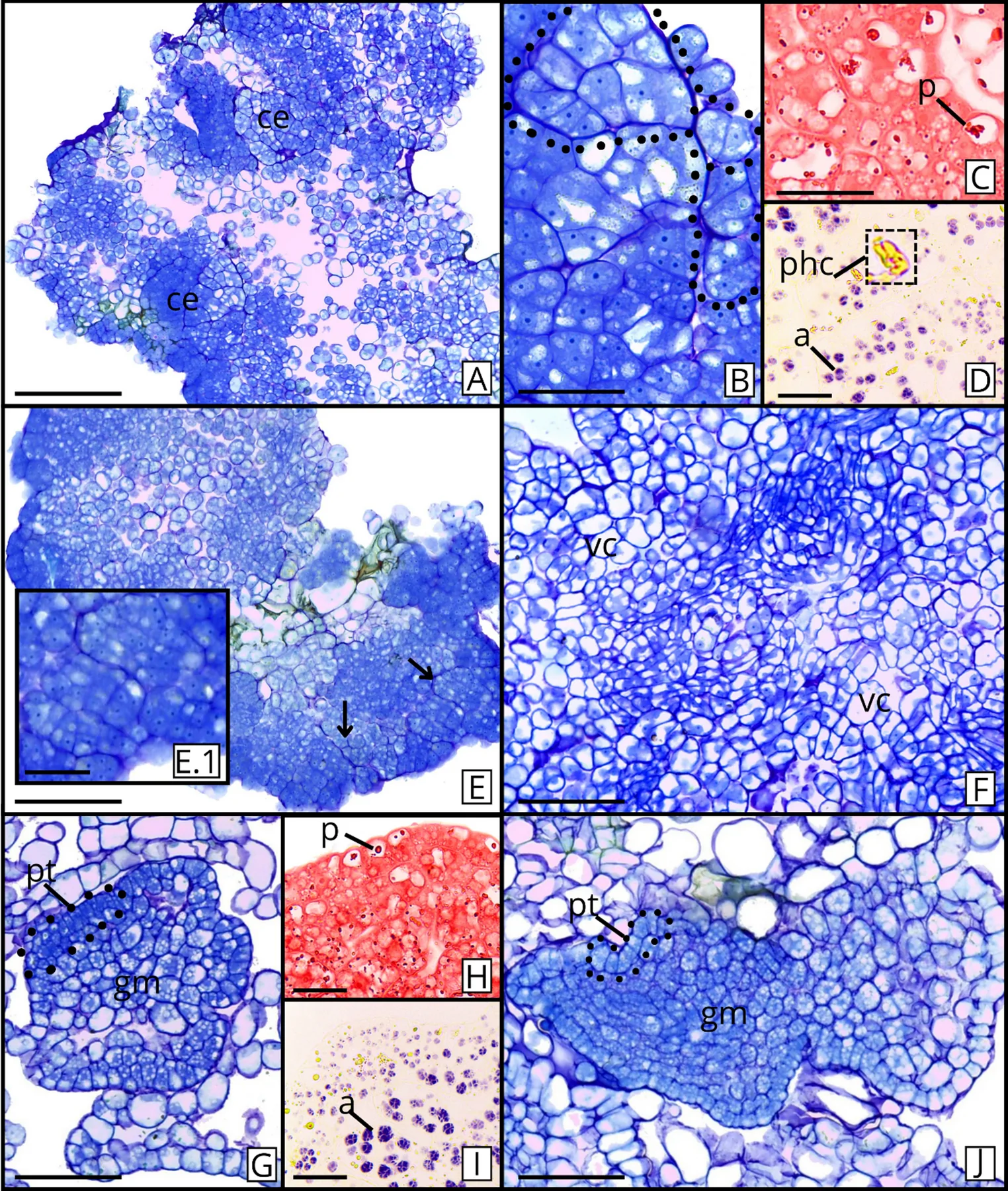
Anatomical and histochemical characteristics of embryogenic calli (45 and 60 days of culture on callus induction medium) and somatic embryos at the globular and torpedo stages (30 days on embryo differentiation medium) of *Piper aduncum*. (**A**) Embryogenic callus with friable morphology and wide intercellular spaces. (**B**) Formation of proembryos (dashed outlines) characterized by cell divisions forming clusters of 2–5 cells. (**C**) Xylidine Ponceau staining showing protein bodies and intense cytoplasmic coloration. (**D**) Starch accumulation and presence of phenolic compounds (dashed square). (**E**) Groups of meristematic cells showing signs of isolation (arrows). (**E**.1) Detail of the cell isolation process observed in meristematic clusters. (**f**) Vacuolated cells and signs of degeneration after 60 days of culture. (**G**, **J**) Somatic embryos at the globular and torpedo stages, respectively. (**H**) Protein bodies in the protoderm and ground meristem (arrows). (**I**) Starch grains concentrated near the ground meristem. Abbreviations: a, starch; ec, embryogenic callus; gm, ground meristem; p, protein; pe, proembryo; phc, phenolic compound; pt, protoderm; vc, vacuolated cell. Scale bars = 200 μm (**A**, **E**); 50 μm (**B**, **F**, **G**, **J**); 25 μm (**C**, **D**, **E**.1, **H**, **I**)

After 30 days of culture, meristematic proliferation intensified relative to earlier stages (Fig. 5e–f), giving rise to compact embryogenic zones composed of isodiametric cells with dense cytoplasm, centralized nuclei, and prominent nucleoli (Fig. 5e). By day 45, embryogenic calli displayed expanded intercellular spaces consistent with friable morphology (Fig. 6a), while cells retained embryogenic features such as dense cytoplasm, large nuclei, and high nucleus-to-cytoplasm ratio (Verdeil et al. 2001; Silva-Cardoso et al. 2022).

Dividing embryogenic cells formed small clusters of two to thirteen cells (Fig. 6b), characteristic of proembryos arising from unicellular origins (Verdeil et al. 2001; Corredoira et al. 2006; Ferreira et al. 2022). Histochemical staining with Xylidine Ponceau revealed protein bodies in embryogenic regions and developing proembryos, with intense red cytoplasmic staining (Fig. 6c), indicative of high mitotic and biosynthetic activity (Silva-Cardoso et al. 2019; Sousa et al. 2020; Ferreira et al. 2022). Starch and phenolic deposits were also detected (Fig. 6d), suggesting stored energy for active division (Silva-Cardoso et al. 2019; Ferreira et al. 2022).

Compact cell clusters within meristematic regions appeared to detach from surrounding tissue (Fig. 6e–e.1), indicating possible multicellular embryo origins. Similar coexistence of unicellular and multicellular embryogenic routes has been observed in *Theobroma cacao* and other species (Verdeil et al. 2001; Maximova et al. 2002; Fehér 2015), reflecting callus heterogeneity and embryogenic plasticity (Jiménez 2001; Silva-Cardoso et al. 2022). After 60 days on induction medium, vacuolated cells predominated (Fig. 6f), indicating loss of viability due to prolonged auxin exposure and stress (Rose 2019).

#### Embryo differentiation and maturation (≥ 60 days)

After 30 days on differentiation medium containing 2,4-D and BAP, histodifferentiation began with the appearance of globular and torpedo embryos (Fig. 6g, j) showing defined protoderm and ground meristem. Protein bodies localized in the protoderm and ground meristem (Fig. 6h), and starch grains near the ground meristem (Fig. 6i) indicated high metabolic activity.

**Fig. 7:**
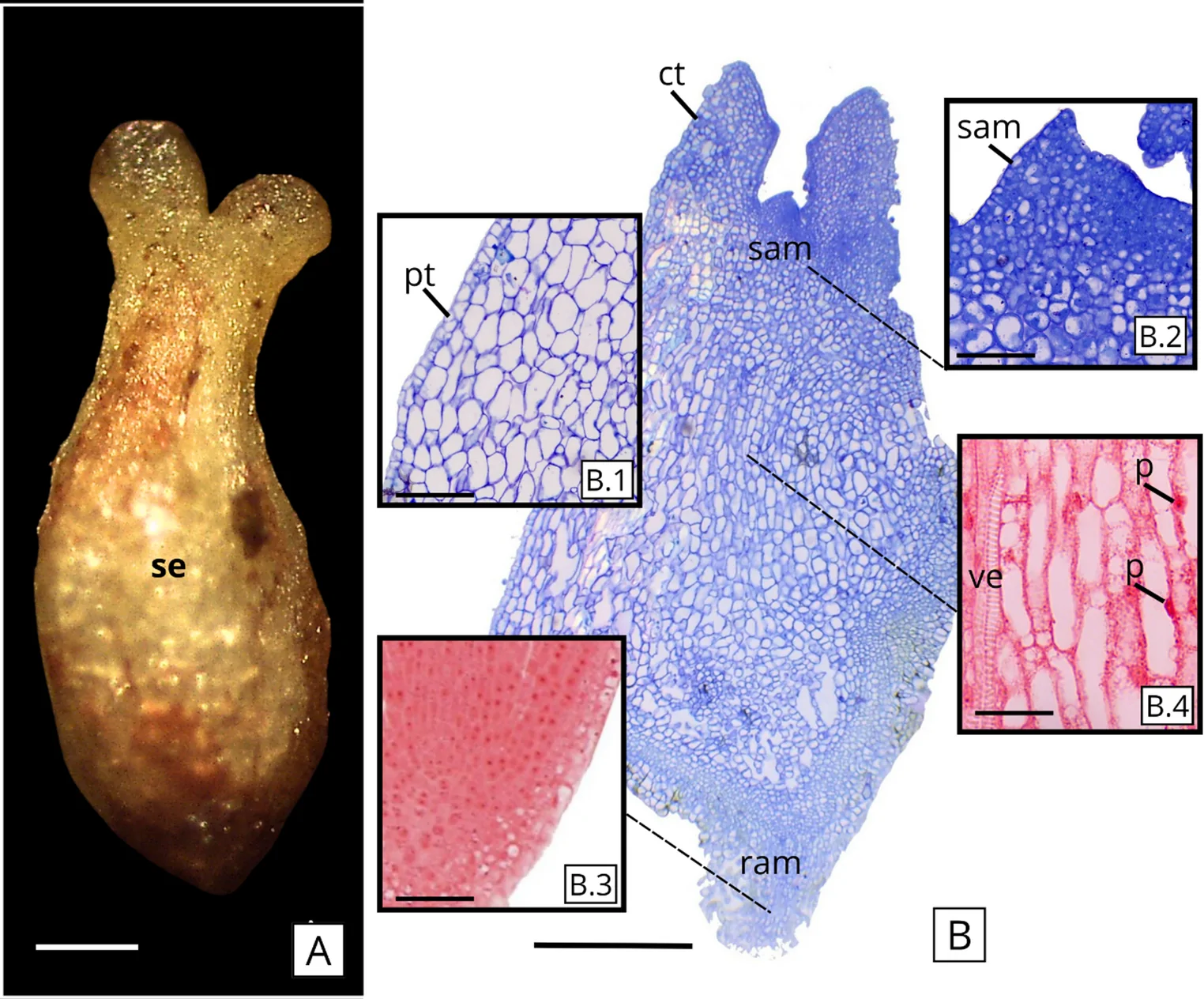
Morphoanatomical and histochemical characterization of a cotyledonary somatic embryo of *Piper aduncum* after 30 days in maturation medium. (**A**) Isolated somatic embryo showing morphology consistent with the cotyledonary stage (note the cotyledons). (**B**) Longitudinal section of the embryo showing the presence of the main primary meristems. (B.1) Protoderm, composed of a single organized cell layer. (B.2) Shoot apical meristem with small, densely arranged cells exhibiting intense meristematic activity. (B.3) Root apical meristem. (B.4) Procambium with vessel elements and residual protein bodies in the ground meristem cells. Abbreviations: ct, cotyledon; pt, protoderm; sam, shoot apical meristem; ram, root apical meristem; pc, procambium; ve, vessel element. Scale bars = 0.5 mm (**A**); 200 μm (**B**); 50 μm (B.1–B.4)

Following 60 days in maturation medium with 45 g L⁻¹ sucrose, embryos developed to the cotyledonary stage (Fig. 7a). Morphoanatomical and histochemical analyses revealed all primary meristems typical of zygotic embryos, including protoderm (Fig. 7b, b.1), procambium (Fig. 7b, b.4), shoot apical meristem (Fig. 7b, b.2), and root apical meristem (Fig. 7b, b.3), demonstrating advanced cellular organization. Although starch was absent, protein accumulation in the ground meristem (Fig. 6b.4), particularly near the procambium and root apical meristem (Fig. 6b.3), confirmed high metabolic activity, as reported for *P. aduncum* (De Sousa et al. 2020). The absence of starch likely reflects intense energy consumption during embryo maturation (Silva-Cardoso et al. 2019; Martins et al. 2022). The presence of protoderm, procambium, and apical meristems attests to the structural equivalence between somatic and zygotic embryos, confirming the successful completion of the embryogenic process under the adopted conditions.

### Callus growth curve in *Piper aduncum*: physiological phases, morphoanatomical correlations, and optimal subculture point

**Fig. 8:**
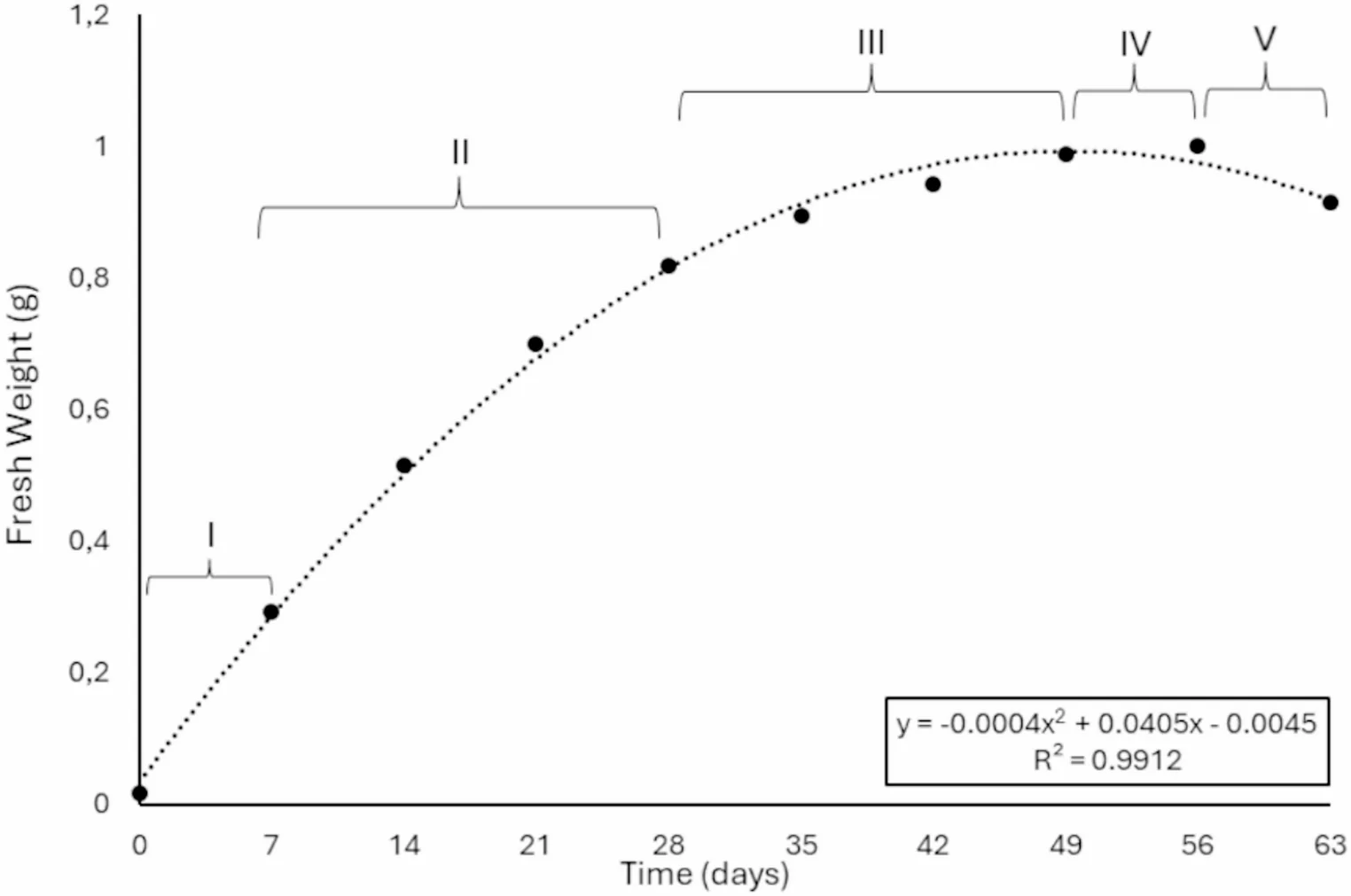
Growth curve of calli formed from leaf explants of *Piper aduncum* cultured on MS medium supplemented with 5 mg·L⁻¹ NAA and 2.5 mg·L⁻¹ BAP over 63 days of cultivation. The curve was fitted by quadratic regression with the equation *y* = − 0.0004*x*² + 0.0405*x* – 0.0045; *R*² = 0.9912). The distinct growth phases correspond to: Phase I – lag (adaptation); Phase II – exponential growth; Phase III – linear; Phase IV – deceleration; Phase V – decline

Growth curves are commonly established to identify physiological phases and determine the optimal timing for subculturing explants into fresh media (Santos et al. 2010). In *P. aduncum*, the growth curve exhibited a quadratic pattern with downward concavity (x² coefficient = − 0.0004). Variations in fresh mass over 63 days of culture revealed five physiological phases (Fig. 8). The lag phase was characterized by explant adaptation and metabolic activation, resulting in 23.55% cumulative growth. Histological analyses revealed early cell divisions, reduction of intercellular spaces, epidermal mitotic activity, and activation of vascular tissues (Fig. 4b–c). Similar lag phases have been reported in other woody and medicinal species, although their duration may vary according to genotype and culture conditions (Santiago 2003; Stein et al. 2010; Santos et al. 2016; Filho et al. 2023; Silva et al. 2020). The exponential phase represented the highest biomass accumulation (46.4%) and coincided with intense cellular proliferation, mesophyll disorganization, and establishment of meristematic regions associated with embryogenic competence (Fig. 5a–d). Between days 28 and 49, corresponding to the linear phase, embryogenic calli became more evident, showing compact isodiametric cells with dense cytoplasm and formation of proembryos (Fig. 6a–e). These characteristics are commonly associated with active somatic embryogenesis in several species (Stein et al. 2010; Santos et al. 2016; Filho et al. 2023; Silva et al. 2020). From days 49 to 56, the deceleration phase showed minimal biomass increase (1.3%), indicating reduction in cell division rates. Although this phase is traditionally considered appropriate for subculture (Nogueira et al. 2008; Stein et al. 2010; Santos et al. 2016; Filho et al. 2023), morphoanatomical analyses indicated that the preceding linear phase was more suitable for transfer due to the higher embryogenic activity observed during this interval. No stationary phase was detected. Instead, the decline phase (56–63 days) was marked by reduced fresh mass, predominance of vacuolated cells, and structural disorganization, indicating progressive loss of viability in prolonged cultures (Fig. 6d). Altogether, the integration of growth kinetics and anatomical markers enabled the delineation of distinct callogenic developmental phases and the determination of the optimal subculture point for advancing somatic embryogenesis in *P. aduncum.*

### Redox dynamics during callogenesis and embryogenic differentiation in *Piper aduncum*: antioxidant activity and lipid peroxidation

**Fig. 9:**
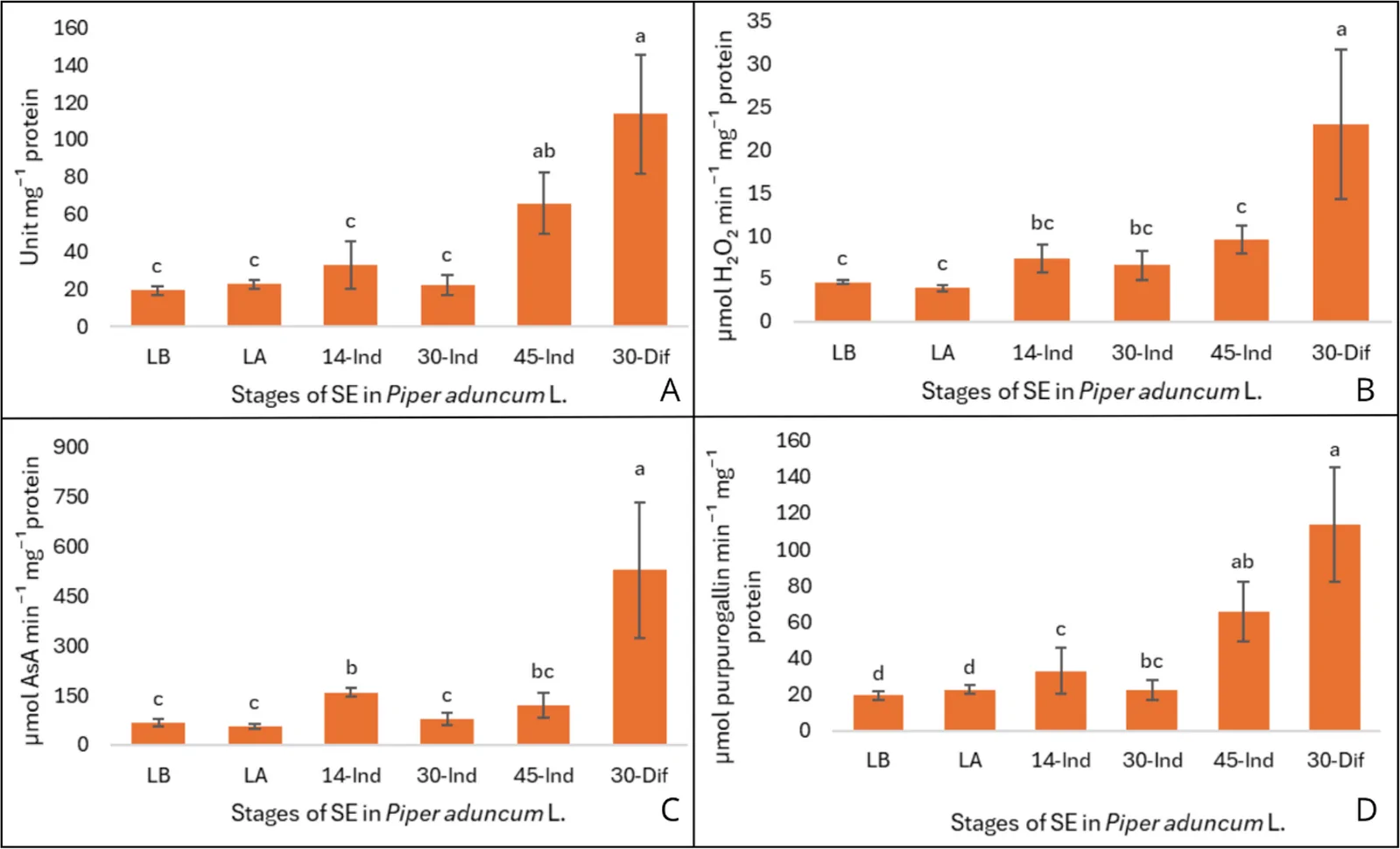
Antioxidant enzyme activities at different stages of somatic embryogenesis in *Piper aduncum* L. (**A**) Superoxide dismutase (SOD), (**B**) Catalase (CAT), (**C**) Ascorbate peroxidase (APX), and (**D**) Peroxidase (POD). LA – leaf before disinfestation; LD – leaf after disinfestation; 14-Ind, 30-Ind, and 45-Ind – calli after 14, 30, and 45 days on induction medium; 30-Dif – calli after 30 days on differentiation medium. Values represent mean ± SE; different letters indicate statistically significant differences according to Tukey’s test (*p* ≤ 0.05)

**Fig. 10:**
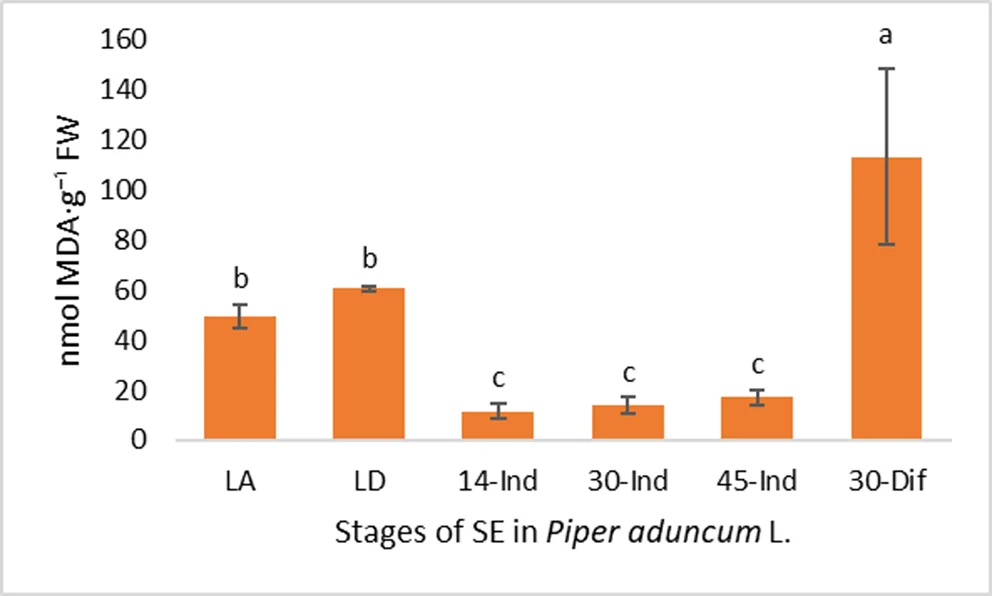
Lipid peroxidation levels, expressed as malondialdehyde (MDA) content, at different stages of somatic embryogenesis in *Piper aduncum* L. LA – leaf before disinfestation; LD – leaf after disinfestation; 14-Ind, 30-Ind, and 45-Ind – calli after 14, 30, and 45 days on induction medium; 30-Dif – calli after 30 days on differentiation medium. Values are presented as mean ± SE; different letters indicate significantly different means according to Tukey’s test (*p* ≤ 0.05)

Antioxidant activity exhibited a convergent pattern among enzymes, with basal levels in leaf tissues, intermediate levels during callus induction, and maximal values during differentiation (Fig. 9a–d). Malondialdehyde (MDA), in turn, increased progressively after 30 days of induction, peaking at the differentiation stage (Fig. 10). No significant differences were observed in enzyme activities in leaf tissues before and after surface sterilization, although a slight 22.6% increase in MDA indicated minor transient stress without lasting physiological effects.

#### Early oxidative activation and competence acquisition (0–14 days)

Superoxide dismutase (SOD) functions as the first defensive barrier, converting superoxide radicals (O₂•⁻) into hydrogen peroxide (H₂O₂), subsequently detoxified by catalase (CAT) and ascorbate peroxidase (APX), with auxiliary action by peroxidase (POD) (Zandi and Schnug 2022). In non-cultured explants, ROS generation was low, maintaining minimal enzyme activity. After 14 days, however, a coordinated activation occurred: SOD and CAT activities increased by 45.4% and 87.6%, respectively, while APX and POD rose by 183.9% and 342.1% (Fig. 9a–d). Concomitant histological analyses revealed cell divisions in mesophyll and epidermis and reactivity in vascular bundles, hallmarks of a controlled oxidative stress peak, where moderate H₂O₂ accumulation signals reprogramming and competence acquisition without membrane damage (Mittler 2002; Fatima et al. 2011; Das and Roychoudhury 2014). Comparable transient ROS elevations have been described as morphogenetic triggers of somatic embryogenesis when accompanied by balanced antioxidant responses (Cui et al. 1999; Libik et al. 2005; Fatima et al. 2011).

#### Enzymatic depression and permissive redox window (14–30 days)

Although SOD and CAT activities continued to increase globally as embryogenesis advanced (Fig. 9a–b), a temporary reduction occurred at 30 days, with decreases of 32.2% for SOD, 10.8% for CAT, and 50.8% for APX, while MDA remained low and statistically unchanged between 14 and 30 days (11.59–13.97 nmol MDA g⁻¹ FW).This enzymatic “valley,” coinciding with the emergence of meristematic zones and proembryos, reflects a permissive ROS window, in which reduced detoxification permits localized H₂O₂ accumulation sufficient to consolidate embryogenic identity without enhancing lipid peroxidation. The literature emphasizes this fine balance between signal (low–moderate H₂O₂) and damage, as well as the spatial control of redox homeostasis in embryogenic calli (Libik et al. 2005; Fatima et al. 2011; Zandi and Schnug 2022).

#### Secondary oxidative burst and redox compensation (30–45 days)

By 45 days, all antioxidant enzymes reached renewed peaks, accompanied by higher MDA levels (17.13 µmol g⁻¹ FM), suggesting a second oxidative wave associated with elevated metabolic activity in proembryogenic calli undergoing linearization and differentiation. This combination of intense ROS generation followed by compensatory antioxidant activation is a common regulatory mechanism in embryogenic systems (Cui et al. 1999; Fatima et al. 2011; Bezirganoglu et al. 2023).

#### Differentiation phase: high oxidative stress and enzymatic stabilization

During the differentiation phase, the highest antioxidant and MDA levels were observed, indicating that somatic embryo formation and maturation impose intense oxidative stress, eliciting maximal antioxidant defense. The marked increase in SOD activity suggests high H₂O₂ production in developing embryos, likely due to rapid proliferation, tissue differentiation, and storage compound synthesis, all processes that elevate O₂ consumption and ROS generation (Fatima et al. 2011; Zandi and Schnug 2022).

Although some studies have reported reduced CAT and APX activity during differentiation as favorable for H₂O₂ signaling (Cui et al. 1999; Libik et al. 2005; Fatima et al. 2011; Bezirganoglu et al. 2023), in *P. aduncum* all antioxidant enzymes remained highly active. Once embryogenic identity is consolidated, excess H₂O₂ becomes detrimental; thus, elevated antioxidant activity safeguards cellular integrity and embryo survival.

#### A model of stress-assisted embryogenesis

Altogether, the results support a stress-assisted somatic embryogenesis model, in which transient ROS pulses function as morphogenetic signals and the antioxidant system defines the threshold between competence, differentiation, and oxidative injury. Recent evidence aligns with this model: ROS effects are dose- and compartment-dependent, with permissive windows enabling reprogramming and damaging ones emerging when buffering capacity fails (Ali and Muday 2024). Redox modulation by ascorbic acid or brassinosteroids enhances embryogenesis efficiency (↑SOD/CAT, ↓MDA) (Hazubska-Przybył et al. 2024), and redox-regulated cellular processes such as autophagy play critical roles in embryogenic progression (Gao et al. 2025). Similar principles govern microsporogenesis, where controlled ROS balance enhances embryo yield, whereas oxidative excess induces cell death (Rueda-Varela et al. 2025).

## Conclusion

Morphoanatomical, histochemical, and physiological analyses demonstrated that somatic embryogenesis in *Piper aduncum* can be initiated from cells associated with vascular bundles, which act as preferential niches for cellular reprogramming, even in adult tissues derived from *ex vitro*-grown plants. The developmental sequence observed, including proembryos, globular, torpedo, and cotyledonary stages, exhibited structural organization comparable to that of zygotic embryos, confirming the morphogenetic competence of the system. The integration of the callus growth curve with anatomical analyses indicated that the linear phase (28–49 days) represents the most suitable subculture point, coinciding with the emergence of embryogenic structures and preceding the decline in viability seen at later stages. In parallel, the profile of antioxidant enzymes and lipid peroxidation revealed that transient oxidative stress pulses, modulated by antioxidant activity, act as key signals for competence acquisition and embryogenic differentiation. Altogether, these findings provide new insights into the cellular and physiological mechanisms underlying somatic embryogenesis in *P. aduncum* and establish practical parameters for optimizing clonal propagation and conservation of elite genotypes with agronomic value.

## Data Availability

The datasets generated and/or analyzed during the current study are available from the corresponding author upon reasonable request.

## References

[CR1] Ahmad P, Jaleel CA, Salem MA et al (2010) Roles of enzymatic and nonenzymatic antioxidants in plants during abiotic stress. Crit Rev Biotechnol 30:161–175. 10.3109/07388550903524243 20214435 10.3109/07388550903524243

[CR2] Ali MF, Muday GK (2024) Reactive oxygen species are signaling molecules that modulate plant reproduction. Plant Cell Environ 47(5):1592–1605. 10.1111/pce.14837 38282262 10.1111/pce.14837

[CR91] Alison MR, Poulsom R, Forbes S, Wright NA (2002) An introduction to stem cells. J Pathol 197:419–423 10.1002/path.1187 12115858

[CR114] Azevedo RA, Alas RM, Smith RJ, Lea PJ (1998) Response of antioxidant enzymes to transfer from elevated carbon dioxide to air and ozone fumigation, in the leaves and roots of wild-type and a catalase-deficient mutant of barley. Physiol Plant 104:280–292. 10.1034/j.1399-3054.1998.1040217.x

[CR3] Ballester A, Corredoira E, Vieitez A (2016) Limitations of somatic embryogenesis in hardwood trees. In: Park YS, Bonga JM, Moon HK (eds) Vegetative Propagation of Forest Trees. National Institute of Forest Science (NiFos), Seoul, Republic of Korea, pp 56–74

[CR4] Batool A, Akram NA, Cheng ZG, L GC et al (2019) Physiological and biochemical responses of two spring wheat genotypes to non-hydraulic root-to-shoot signalling of partial and full root-zone drought stress. Plant Sci 153(1):65–72. 10.1016/j.plaphy.2019.03.001 10.1016/j.plaphy.2019.03.001 30875531

[CR109] Beauchamp C, Fridovich I (1971) Superoxide dismutase: improved assays and an assay applicable to acrylamide gels. Anal Biochem 44:276–287. 10.1016/0003-2697(71)90370-8 10.1016/0003-2697(71)90370-8 4943714

[CR5] Berlyn GP, Miksche JP, Sass JE (1976) Botanical microtechnique and cytochemistry. Iowa State University, Ames, Iowa

[CR108] Bezirganoglu I, Yazıcılar B (2023) Salicylic acid improves somatic embryogenesis system in triticale using mature embryos. Eurasian J Mol Biochem Sci 2:14–18. https://izlik.org/JA43UH33MS

[CR6] Campos-Boza S, Vinas M, Solórzano-Cascante P, Holst A, Steinmacher DA, Guerra MP, Jiménez VM (2022) Somatic embryogenesis and plant regeneration from transverse thin cell layers of adult peach palm (*Bactris gasipaes*) lateral offshoots. Front Plant Sci 13. 10.3389/fpls.2022.995307 10.3389/fpls.2022.995307 PMC9554471 36247585

[CR7] Cao X, Gao F, Qin C, Chen S et al (2022) Optimizing Somatic Embryogenesis Initiation, Maturation and Preculturing for Cryopreservation in *Picea pungens*. Forests 13(12):2097. 10.3390/f13122097

[CR8] Capriotti L, Limera C, Mezzetti B et al (2022) From induction to embryo proliferation: improved somatic embryogenesis protocol in grapevine for Italian cultivars and hybrid Vitis rootstocks. Plant Cell Tiss Organ Cult 151:221–233. 10.1007/s11240-022-02346-w

[CR9] Carvalho JMFC, Lima MMA, Aires PSR, Vidal MS, Pimentel NW (2006) Embriogênese Somática. Embrapa Algodão. https://www.infoteca.cnptia.embrapa.br/infoteca/handle/doc/276541. Accessed 08 Jul 2025

[CR10] Cid LP, Teixeira JB (2010) Explante, meio nutritivo, luz e temperatura. In: Cid LP (org) Cultivo *in vitro* de plantas. Embrapa Informação Tecnológica, Brasília, DF, pp 15–30

[CR11] Corredoira E, Merkle SA, Martínez MT et al (2019) Non-Zygotic Embryogenesis in Hardwood Species. CRC Crit Rev Plant Sci 38(1):29–97. 10.1080/07352689.2018.1551122

[CR12] Corredoira E, Valladares S, Vieitez AM (2006) Morphohistological analysis of the origin and development of somatic embryos from leaves of mature Quercus robur. Vitro Cell Dev Biol -Plant 42:525–533. 10.1079/IVP2006827

[CR106] Cui K, Xing G, Liu X, Xing G, Wang Y (1999) Effect of hydrogen peroxide on somatic embryogenesis of *Lycium barbarum* L. Plant Sci 146:9–16. 10.1016/S0168-9452(99)00087-4

[CR13] De Paiva Neto VB, Otoni WC (2003) Carbon sources and their osmotic potential in plant tissue culture: does it matter? Sci Hortic (Amsterdam) 97:193–202. 10.1016/S0304-4238(02)00231-5

[CR15] De Sousa PCA, Souza SSSE, Meira FS, Meira RO, Gomes HT, Silva-Cardoso IMA, Scherwinski-Pereira JE (2020) Somatic embryogenesis and plant regeneration in *Piper aduncum* L. Vitro Cell Dev Biol -Plant 56:618–633. 10.1007/s11627-020-10110-y

[CR14] De Sousa PCA, Souza SSS, Nogueira GF, Silva-Cardoso IMA, Scherwinski-Pereira JE (2022) Indirect somatic embryogenesis of *Piper hispidinervum* L. and evaluation of the regenerated plants by flow cytometry. J Genet Eng Biotechnol 20(1):40. 10.1186/s43141-022-00323-6 35230554 10.1186/s43141-022-00323-6 PMC8888786

[CR16] Dousseau S (2009) Propagação, características fotossintéticas, estruturais, fitoquímicas e crescimento inicial de *Piper aduncum* L (Piperaceae). Dissertation, Universidade Federal de Lavras

[CR17] Durofil A, Radice M, Blanco-Salas J, Ruiz-Téllez T (2021) *Piper aduncum* essential oil: a promising insecticide, acaricide and antiparasitic. A review. Parasite 28:42. 10.1051/parasite/2021040 33944775 10.1051/parasite/2021040 PMC8095093

[CR18] Elhiti M, Stasolla C (2022) Transduction of Signals during Somatic Embryogenesis. Plants 11(2):178. 10.3390/plants11020178 35050066 10.3390/plants11020178 PMC8779037

[CR19] Espinoza PEV, Benítez-García I, Peralta ALL, Paredes-López O, Villar-Martínez AAD (2017) Somatic embryogenesis from leaf explants of *Tagetes erecta* L. Plant Biotechnol 34:187–192. 10.5511/plantbiotechnology.17.1120a 10.5511/plantbiotechnology.17.1120a PMC6543694 31275026

[CR105] Fatima N, Ahmad N, Anis M (2011) Enhanced in vitro regeneration and change in photosynthetic pigments, biomass and proline content in *Withania somnifera* L. induced by copper and zinc ions. Plant Physiol Biochem 49:1465–1471. 10.1016/j.plaphy.2011.08.011 10.1016/j.plaphy.2011.08.011 22078385

[CR20] Fazolin M, Bizzo HR, Monteiro AFM, Lima MEC, Maisforte NS, Gama PE (2023) Synergism in Two-Component Insecticides with dillapiole against Fall Armyworm. Plants 12:3042. 10.3390/plants12173042 37687289 10.3390/plants12173042 PMC10489978

[CR21] Fazolin M, Estrela JL, Catani V, Lima MSD, Alécio MR (2005) Toxicidade do óleo de *Piper aduncum* L. a adultos de *Cerotoma tingomarianus* Bechyné (Coleoptera: Chrysomelidae). Neotrop Entomol 34:485–489. 10.1590/S1519-566X2005000300018

[CR22] Fazolin M, Estrela JLV, Catani V, Alécio MR, Lima MS (2007) Propriedade inseticida dos óleos essenciais de *Piper hispidinervum* C. DC.; *Piper aduncum* L. e *Tanaecium nocturnum* (Barb. Rodr.) Bur. & K. Shum sobre *Tenebrio molitor* L., 1758. Ciênc Agrotec 31:113–120. 10.1590/S1413-70542007000100017

[CR23] Fazolin M, Estrela JLV, Catani V, Costa CR (2006) Potencialidades da pimenta-de-macaco (*Piper aduncum* L.): características gerais e resultados de pesquisa. Embrapa Acre, Rio Branco

[CR24] Fazolin M, Estrela JLV, Monteiro AFM, Gomes LP, Silva IM, Silva MSF (2015) Sinérgico alternativo para o manejo da resistência da lagarta-do-cartucho do milho a piretróides. Revista Brasileira de Milho e Sorgo 14:316–325. 10.18512/1980-6477/rbms.v14n3p316-325

[CR25] Fazolin M, Monteiro AFM, Malavazi FW, Estrela JLV (2022) Óleos Essenciais Como Sinergistas de Formulações Inseticidas. In: Ribeiro RP, Vendramim JD, Baldin ELL (eds) Inseticidas botânicos no Brasil: aplicações, potencialidades e perspectivas. FEALQ, Piracicaba, pp 557–615. *In*

[CR94] Fehér A (2005) Why somatic plant cells start to form embryos? In: Mujib A, Šamaj J (eds) Somatic embryogenesis. Plant Cell Monographs, vol 2. Springer, Berlin, Heidelberg. 10.1007/7089_019

[CR26] Fehér A (2015) Somatic embryogenesis—Stress-induced remodeling of plant cell fate. Biochim Biophys Acta 1849:385–402. 10.1016/j.bbagrm.2014.07.005 25038583 10.1016/j.bbagrm.2014.07.005

[CR27] Fehér A (2019) Callus, Dedifferentiation, Totipotency, Somatic Embryogenesis: What These Terms Mean in the Era of Molecular Plant Biology? Front Plant Sci 10:442509. 10.3389/fpls.2019.00536 10.3389/fpls.2019.00536 PMC6524723 31134106

[CR28] Ferrari IF, Marques GA, Junior WLS et al (2021) Comparative ontogenesis of *Coffea arabica* L. somatic embryos reveals the efficiency of regeneration modulated by the explant source and the embryogenesis pathway. *In Vitro* Cell Dev Biol Plant 57:796–810. 10.1007/s11627-021-10200-5

[CR29] Ferreira JCB, Silva-Cardoso IMA, Meira RO, Scherwinski-Pereira JE (2022) Somatic embryogenesis and plant regeneration from zygotic embryos of the palm tree *Euterpe precatoria* Mart. Plant Cell Tiss Organ Cult 148:667–686. 10.1007/s11240-022-02227-2

[CR30] Ferriani AP, Gomes EN, Krinski D, Deschamps C (2019) Vegetative propagation of *Piper aduncum* L. (matico) using cuttings of varying lengths and different substrates. Rev Cubana Plantas Medicinales 23:3

[CR101] Filho RSLC, Silva TS, Ribeiro YRS, Santa-Catarina C, Santana JRF (2023) Caracterização histomorfológica e bioquímica de calos induzidos em *Cenostigma pyramidale* [Tul.] Gagnon & G.P. Lewis. Cienc Florest 33:e66334

[CR31] Gao E, Zhao Y, Wu M, Wang K, Zheng Q, Li Y, Qu X, Wu X, Guo W, Wang P (2025) Autophagy is essential for somatic embryogenesis in citrus through regulating amyloplast degradation and lipid homeostasis. New Phytol 245:684–697. 10.1111/nph.20242 39497370 10.1111/nph.20242

[CR32] Garcia C, de Almeida AAF, Costa M et al (2019) Abnormalities in somatic embryogenesis caused by 2,4-D: an overview. Plant Cell Tiss Organ Cult 137:193–212. 10.1007/s11240-019-01569-8

[CR33] George EF, Hall MA, Klerk GJ (2008) The components of plant tissue culture media II: Organic additions, osmotic and pH effects, and support systems. In: George EF, Hall MA, Klerk GJ (eds) Plant propagation by tissue culture. Springer Netherlands, Dordrecht, pp 115–173. 10.1007/978-1-4020-5005-3_4

[CR34] Gomes EN, Krinski D (2018) Rooting of apical, median and basal stem cuttings of *Piper aduncum* L. on different substrates. Rev Ciênc Agrovet 17:435. 10.5965/223811711732018435

[CR87] Guimarães MCM (2015) Estudo da calogênese, dinâmica de crescimento de calos e estabelecimento de suspensões celulares de *Piper permucronatum*. Dissertation, Universidade Federal de Rondônia, Porto Velho

[CR35] Guo F, Wang H, Lian G et al (2023) Initiation of scutellum-derived callus is regulated by an embryo-like developmental pathway in rice. Commun Biol 6:457. 10.1038/s42003-023-04835-w 10.1038/s42003-023-04835-w PMC10130139 37100819

[CR112] Havir EA, McHale NA (1987) Biochemical and developmental characterization of multiple forms of catalase in tobacco leaves. Plant Physiol 84:450–455. 10.1104/pp.84.2.450 10.1104/pp.84.2.450 PMC1056601 16665461

[CR36] Hazubska-Przybył T, Obarska A, Konecka A, Wawrzyniak MK et al (2024) Modulating ascorbic acid levels to optimize somatic embryogenesis in *Picea abies* (L.) H. Karst. Insights into oxidative stress and endogenous phytohormones regulation. Front Plant Sci 15:1372764. 10.3389/fpls.2024.1372764 38903446 10.3389/fpls.2024.1372764 PMC11188323

[CR90] Hiroo F (2004) Signals that control plant vascular cell differentiation. Nat Rev Mol Cell Biol 5(5):379–391. 10.1038/nrm1364 10.1038/nrm1364 15122351

[CR37] Horstman A, Bemer M, Boutilier K (2017) A transcriptional view on somatic embryogenesis. Regeneration 4(4):201. 10.1002/reg2.91 29299323 10.1002/reg2.91 PMC5743784

[CR38] Jiménez VM (2001) Regulation of in vitro somatic embryogenesis with emphasis on to the role of endogenous hormones. Rev Bras Fisiol Veg 13:2. 10.1590/S0103-31312001000200008

[CR85] Judd WS, Campbell CS, Kellogg EA, Stevens PF, Cantino PD (1999). Plant systematics. a phylogenetic approach. Systematic Biology, 48(4):826–828

[CR39] Karami O, Jong H, Somovilla VJ et al (2023) Structure–activity relationship of 2,4-D correlates auxinic activity with the induction of somatic embryogenesis in *Arabidopsis thaliana*. Plant J 116(5):1355–1369. 10.1111/tpj.16430 37647363 10.1111/tpj.16430

[CR81] Karnovsky MJA (1965) Formaldehyde-glutaraldeyde fixative of high osmolality for use in electron microscopy. J Cell Biol 27:137–138

[CR40] Knörzer OC, Burner J, Boger P (1996) Alterations in the antioxidative system of suspension-cultured soybean cells (Glycine max) induced by oxidative stress. Physiol Plant 97(2). 10.1034/j.1399-3054.1996.970225.x

[CR107] Libik M, Konieczny R, Pater B et al (2005) Differences in the activities of some antioxidant enzymes and in H₂O₂ content during rhizogenesis and somatic embryogenesis in callus cultures of the ice plant. Plant Cell Rep 23:834–841. 10.1007/s00299-004-0886-8 10.1007/s00299-004-0886-8 15517278

[CR41] Li M, Heidmann I, Horstman A, Chen B et al (2022) Auxin biosynthesis maintains embryo identity and growth during BABY BOOM-induced somatic embryogenesis. Plant Physiol 188(2):1095–1110. 10.1093/plphys/kiab558 34865162 10.1093/plphys/kiab558 PMC8825264

[CR42] Luo Q, Hu S, Deng Z et al (2024) Plant peptide hormone phytosulfokine promotes embryo development of mass in *Pinus massoniana*. Plant Cell Tiss Organ Cult 158:58. 10.1007/s11240-024-02857-8

[CR43] Maciel ALDR, Pasqual M, Pereira AR et al (2003) Embriogênese somática indireta em explantes foliares de *Coffea arabica* L. CV. Obatã Ciênc agrotec 27(1). 10.1590/S1413-70542003000100013

[CR86] Maciel SA, Teixeira RB, Raposo A, Fermino Junior PCP (2014) Anatomia comparada de folhas de pimenta longa (*Piper hispidinervum* C. DC.) e pimenta de macaco (*Piper aduncum* L.) cultivadas in vitro, ex vitro e in vivo. Biotemas 27:11–19

[CR93] Mahonen AP, Bishopp A, Higuchi M, Nieminen KM, Kinoshita K, Tormakangas K, Ikeda Y, Oka A, Kakimoto T, Helariutta Y (2006) Cytokinin signaling and its inhibitor AHP6 regulate cell fate during vascular development. Science 311:94–98 10.1126/science.1118875 16400151

[CR44] Martini AN, Papafotiou M, Vemmos SN (2013) Season and Explant Origin Affect Phenolic Content, Browning of Explants, and Micropropagation of × *Malosorbus florentina*. (Zucc) Browicz HortScience 48(1):102. 10.21273/hortsci.48.1.102

[CR45] Martins J, Correia S, Pinto G, Canhoto J (2022) Cloning adult trees of *Arbutus unedo* L. through somatic embryogenesis. Plant Cell Tiss Organ Cult 150:611–626. 10.1007/s11240-022-02314-4

[CR46] Maximova SN, Alemanno L, Young A et al (2002) Efficiency, genotypic variability, and cellular origin of primary and secondary somatic embryogenesis of *Theobroma cacao* L. Vitro Cell Dev Biol —Plant 38:252–259. 10.1079/IVP2001257

[CR47] Meena NL, Kumari A, Maheshwari C, Bhardwaj R, Dhaka AS, Hasan M (2024) Antioxidant Defense Mechanism and High-Temperature Stress Tolerance in Plants. In: Shahid M, Gaur R (eds) Molecular Dynamics of Plant Stress and its Management. Springer, Singapore. 10.1007/978-981-97-1699-9_8

[CR113] Meira FS, Luis ZG, Araújo Silva-Cardoso IM, Scherwinski-Pereira JE (2019) Developmental pathway of somatic embryogenesis from leaf tissues of macaw palm (*Acrocomia aculeata*) revealed by histological events. Flora 250:59–67. 10.1016/j.flora.2018.11.011

[CR110] Meira RO, Joane, Neves JS, Inaê Mariê, Silva-Cardoso IMA, Cunha RNV, Lopes R, de Souza ALX, Scherwinski J (2025) Dual-phase culture system as a promising strategy to enhance the efficiency of somatic embryogenesis and plant regeneration in oil palm (*Elaeis oleifera* × *E. guineensis*) Plant Cell Tissue and Organ Culture (PCTOC) 162(2). 10.1007/s11240-025-03154-8

[CR49] Merkle SA (1995) Strategies for dealing with limitations of somatic embryogenesis in hardwood trees. *In Vitro* Cell Dev Biol A 31:6

[CR104] Mittler R (2002) Oxidative stress, antioxidants and stress tolerance. Trends Plant Sci 7:405–410. 10.1016/S1360-1385(02)02312-9 10.1016/s1360-1385(02)02312-9 12234732

[CR83] Montero-Cortés MI, Chan-Rodríguez JL, Cordova-Lara I, Oropeza-Salin C, Sáenz-Carbonell L (2011) Addition of benzyladenine to coconut explants cultured in vitro improves the formation of somatic embryos and their germination. Agrociencia 45:863–872

[CR80] Murashige T, Skoog F (1962) A revised medium for rapid growth and bio assays with tobacco tissue cultures. Physiol Plant 15:473–497

[CR84] Nakamura AT, Simão E, Silva L, Torres GA (2015) Origin of the subepidermal tissue in *Piper* L. leaves. Braz J Biol 75:744–751 10.1590/1519-6984.13713 26132020

[CR111] Nakano Y, Asada K (1981) Hydrogen peroxide is scavenged by ascorbate-specific peroxidase in spinach chloroplasts. Plant Cell Physiol 22:867–880. 10.1093/oxfordjournals.pcp.a076232

[CR50] Nogueira R, Paiva R, Lima EC et al (2008) Curva de crescimento e análises bioquímicas de calos de murici-pequeno (*Byrsonima intermedia* A. Juss). Rev Bras Pl Med 10(1):44–48

[CR51] Nolan KE, Saeed NA, Rose RJ (2006) The stress kinase gene MtSK1 in *Medicago truncatula* with particular reference to somatic embryogenesis. Plant Cell Rep 25:711–722. 10.1007/s00299-006-0135-4 16518633 10.1007/s00299-006-0135-4

[CR82] O’Brien TP, Feder N, McCully ME (1964) Polychromatic staining of plant cell walls by toluidine blue O. Protoplasma 59:368–373

[CR53] Orłowska A, Kępczyńska E (2020) Oxidative status in *Medicago truncatula* Gaertn. non-embryogenic and embryogenic tissues with particular reference to somatic embryogenesis. Plant Cell Tiss Organ Cult 140:35–48. 10.1007/s11240-019-01709-0

[CR54] Park J, Choi Y, Jeong M et al (2023) Uncovering transcriptional reprogramming during callus development in soybean: Insights and implications. Front Plant Sci 14:1239917. 10.3389/fpls.2023.1239917 37600197 10.3389/fpls.2023.1239917 PMC10436568

[CR95] Pinheiro MVM, Silva TCR, Maia C, Lima BV, Motoike SY (2012) Propagação in vitro de genótipos de alface via embriogênese somática. Cienc Rural 42:1947–1953

[CR55] Ponnusamy B, Staden J (2012) Somatic embryogenesis of *Merwilla plumbea* (Lindl.) Speta. Plant Cell Tiss Organ Cult 109:517–524. 10.1007/s11240-012-0118-9

[CR56] Ramos YJ, Felisberto JS, Gouvêa-Silva JG et al (2022) Phenoplasticity of Essential Oils from Two Species of *Piper* (Piperaceae): Comparing Wild Specimens and Bi-Generational Monoclonal Cultivars. Plants (Basel) 11:1771. 10.3390/plants11131771 35807723 10.3390/plants11131771 PMC9269527

[CR92] Rippon HJ, Bishop AE (2004) Embryonic stem cells. Cell Prolif 37:23–34 10.1111/j.1365-2184.2004.00298.x PMC6495712 14871235

[CR57] Rose RJ (2016) Genetic reprogramming of plant cells in vitro via dedifferentiationor pre-existing stem cells. In: Rose RJ (ed) Molecular cell biology of the growth and differentiation of plant cells. CRC, Boca Raton, pp 320–339

[CR58] Rose RJ (2019) Somatic Embryogenesis in the *Medicago truncatula* Model: Cellular and Molecular Mechanisms. Front Plant Sci 10:267. 10.3389/fpls.2019.00267 30984208 10.3389/fpls.2019.00267 PMC6447896

[CR59] Rueda-Varela C, Carneros E, Caro E et al (2025) Enhancing microspore embryogenesis initiation by reducing ROS, autophagy, and cell death with novel small molecules in rapeseed and barley. J Plant Physiol 311:154546. 10.1016/j.jplph.2025.154546 40527111 10.1016/j.jplph.2025.154546

[CR60] Saleh EOL, Luis ZG, Silva-Cardoso IMA, Scherwinski-Pereira JE (2024) Somatic embryogenesis and plant regeneration from zygotic embryos of babassu (*Attalea speciosa* Mart. ex Spreng), a multi-purpose palm tree. Plant Cell Tiss Organ Cult 159:15. 10.1007/s11240-024-02884-5

[CR61] Sané D, Aberlenc-Bertossi F, Gassama-Dia YK, Sagna M, Trouslot MF, Duval Y, Borgel A (2006) Ultrastructural Changes in *Coconut Calli* Associated with the Acquisition of Embryogenic Competence. Ann Botany 98:301–308. 10.1093/aob/mcl104 16740588 10.1093/aob/mcl104 PMC2803457

[CR98] Santiago EJA Caracterização morfológica e bioquímica de calos de pimenta longa (*Piper hispidinervium* Candolle, De Candolle). 2003. 183 f. Tese (Doutorado), Universidade Federal de Lavras, Lavras

[CR100] Santos MRA, Guimarães MCM, Paz ES, Magalhães GMO, Souza CA, Smozinski CV, Nogueira WO (2016) Induction and growth pattern of callus from *Piper permucronatum* leaves. Rev Bras Plantas Med 18:142–148

[CR88] Santos MRA, Nogueira WO (2018) Calogênese em explantes foliares de *Piper tuberculatum*. South Am J Basic Educ Tech Technol 5:1–10

[CR63] Silva-Cardoso IMA, Gomes ACMM, Scherwinski-Pereira JE (2022) Cellular responses of oil palm genotypes during somatic embryogenesis involve participation of procambial cells, DNA demethylation, and auxin accumulation. Plant Cell Rep 41:1875–1893. 10.1007/s00299-022-02898-3 35776139 10.1007/s00299-022-02898-3

[CR64] Silva-Cardoso IMA, Meira FS, Gomes ACMM, Scherwinski-Pereira JE (2019) Anatomical and histochemical studies of thesomatic embryogenesis of *Syagrus oleracea* from immature inflorescences. Crop Breed Appl Biotechnol 19:444–450. 10.1590/1984-70332019v19n4n62

[CR102] Silva TS, Carvalho Filho RSL, Tanan TT, Rocha TC, Santana JRF (2020) Calogênese em *Myracrodruon urundeuva* Fr. All. Cienc Florest 30:700–717. 10.5902/1980509831689

[CR89] Slesak H, Popielarska M, Góralski G (2005) Morphological and histological aspects of 2,4-D effects on rape explants (*Brassica napus* L. cv. Kana) cultured in vitro. Acta Biol Cracov Ser Bot 47:219–226

[CR65] Souza VC, Lorenzi H (2012) Botânica Sistemática: guia ilustrado para identificação das famílias de Fanerógamas nativas e exóticas no Brasil, baseado em APG III. Dissertation, Universidade de São Paulo

[CR99] Stein VC, Paiva R, Herrera RC, Vargas DP (2010) Division index of *Inga vera* Willd. subsp. affinis (DC.) T.D. Penn. calluses. Rev Cienc Agrar 53:159–163

[CR66] Sugimoto K, Gordon SP, Meyerowitz EM (2011) Regeneration in plants and animals: dedifferentiation, transdifferentiation, or just differentiation? Trends Cell Biol 21:212–218 21236679 10.1016/j.tcb.2010.12.004

[CR67] Susanto D, Sudrajat, Suwinarti W, Amirta R (2018) Seed germination and cuttings growth of *Piper aduncum*. IOP Conf Ser Earth Environ Sci 144:012018. 10.1088/1755-1315/144/1/012018

[CR68] Teisseire H, Guy V (2000) Copper-induced changes in antioxidant enzymes activities in fronds of duckweed (*Lemna minor*). Plant Sci 153(1):65–72. 10.1016/S0168-9452(99)00257-5

[CR69] Thapa CB, Pant KK, Bhattarai HD, Pant B (2024) Indirect somatic embryogenesis and plant regeneration through leaf and nodal cultures of *Piper longum L*. Banko Janakari 34(2):16–28. 10.3126/banko.v34i2.62729

[CR70] Tremblay L, Tremblay FM (1995) Maturation of black spruce somatic embryos: Sucrose hydrolysis and resulting osmotic pressure of the medium. Plant Cell Tiss Organ Cult 42:39–46. 10.1007/BF00037680

[CR71] Ulisses C, Camara TR, Willadino L, Albuquerque CC, Brito JZ (2010) Early Somatic Embryogenesis in *Heliconia chartacea* Lane ex Barreiros cv. Sexy Pink Ovary Section Explants. Braz Arch Biol Technol 53:11–18. 10.1590/S1516-89132010000100002

[CR72] Verdeil JL, Alemanno L, Niemenak N, Trambarger TJ (2007) Pluripotent versus totipotent plant stem cells: dependence versus autonomy? Trends Plant Sci 12:245–252 17499544 10.1016/j.tplants.2007.04.002

[CR73] Verdeil JL, Hocher V, Huet C, Grosdemange F, Escoute J, Ferrière N, Nicole M (2001) Ultrastructural Changes in *Coconut Calli* Associated with the Acquisition of Embryogenic Competence. Ann Botany 88:9–18. 10.1006/anbo.2001.1408

[CR74] Vidal BC (1970) Dichroism in collagen bundles stained with Xylidine-Ponceau 2R. Ann Histochim 15:289–296 4105098

[CR75] Volpe HXL et al (2018) Eficácia do óleo essencial de *Piper aduncum* L. (Piperaceae) para o controle de *Diaphorina citri* Kuwayama. Embrapa Acre, Rio Branco

[CR76] Volpe HXL, Fazolin M, Garcia RB, Magnani RF, Barbosa JC, Miranda MP (2016) Efficacy of essential oil of *Piper aduncum* against nymphs and adults of *Diaphorina citri*. Pest Manag Sci 72:1242–1249. 10.1002/ps.4143 26331551 10.1002/ps.4143

[CR97] von Aderkas P, Bonga JM (2000) Influencing micropropagation and somatic embryogenesis in mature trees by manipulation of phase change, stress and culture environment. Tree Physiol 20:921–928 10.1093/treephys/20.14.921 11303566

[CR77] Wang XD, Nolan KE, Irwanto RR, Sheahan MB, Rose RJ (2011) Ontogeny of embryogenic callus in *Medicago truncatula*: the fate of the pluripotent and totipotent stem cells. Ann Bot 107:599–609. 10.1093/aob/mcq269 10.1093/aob/mcq269 PMC3064535 21224270

[CR103] Zandi P, Schnug E (2022) Reactive oxygen species, antioxidant responses and implications from a microbial modulation perspective. Biology 11:155. 10.3390/biology11020155 10.3390/biology11020155 PMC8869449 35205022

[CR78] Zavattieri MA, Frederico AM, Lima M, Sabino R, Arnholdt-Schmitt B (2010) Induction of somatic embryogenesis as an example of stress-related plant reactions. Electron J Biotechnol 13:1–9. 10.4067/S0717-34582010000100012

[CR79] Zevallos Pérez CA et al (2022) Propiedades farmacológicas e indicaciones terapéuticas de *Piper aduncum* L. (matico) para aliviar diversas enfermedades. Revista de Investigación Universitaria 12:73. 10.53470/riu.v12i1.73

[CR96] Zurita-Valencia W, Gómez-Cruz JE, Atrían-Mendoza E et al (2014) Establecimiento de un método eficiente de germinación in vitro y micropropagación del cirimo (*Tilia mexicana* Schlecht.) (Tiliaceae). Polibotánica 38:129–144

